# The multidrug-resistant PMEN1 pneumococcus is a paradigm for genetic success

**DOI:** 10.1186/gb-2012-13-11-r103

**Published:** 2012-11-16

**Authors:** Kelly L Wyres, Lotte M Lambertsen, Nicholas J Croucher, Lesley McGee, Anne von Gottberg, Josefina Liñares, Michael R Jacobs, Karl G Kristinsson, Bernard W Beall, Keith P Klugman, Julian Parkhill, Regine Hakenbeck, Stephen D Bentley, Angela B Brueggemann

**Affiliations:** 1 Department of Zoology, University of Oxford, South Parks Road, Oxford, OX1 3PS, UK; 2 Department of Microbiology Surveillance and Research, Statens Serum Institut, 5 Artillerivej, DK 2300 Copenhagen S, Denmark; 3 Pathogen Genomics Team, Wellcome Trust Sanger Institute, Wellcome Trust Genome Campus, Hinxton, Cambridge, CB10 1SA, UK; 4 Streptococcus Laboratory, Centers for Disease Control and Prevention, 1600 Clifton Rd, Atlanta, Georgia, 30333, USA; 5 Centre for Respiratory Diseases and Meningitis, National Institute for Communicable Diseases, Sandringham, Johannesburg 2131, South Africa; 6 Department of Microbiology, Bellvitge Hospital-CIBERes-IDIBELL-UB, Feixa Llarga s/n, 08907 Barcelona, Spain; 7 Department of Pathology, Case Western Reserve University, Cleveland, OH 44106, USA; 8 Clinical Microbiology Department, Landspitali University Hospital and University of Iceland, 101 Reykjavik, Iceland; 9 Hubert Department of Global Health Epidemiology, Rollins School of Public Health, 1518 Clifton Road NE, Atlanta, GA 30322, USA; 10 Department of Microbiology, University Kaiserslautern, Paul-Ehrlich-Straße, 67663 Kaiserslautern, Germany

## Abstract

**Background:**

*Streptococcus pneumoniae*, also called the pneumococcus, is a major bacterial pathogen. Since its introduction in the 1940s, penicillin has been the primary treatment for pneumococcal diseases. Penicillin resistance rapidly increased among pneumococci over the past 30 years, and one particular multidrug-resistant clone, PMEN1, became highly prevalent globally. We studied a collection of 426 pneumococci isolated between 1937 and 2007 to better understand the evolution of penicillin resistance within this species.

**Results:**

We discovered that one of the earliest known penicillin-nonsusceptible pneumococci, recovered in 1967 from Australia, was the likely ancestor of PMEN1, since approximately 95% of coding sequences identified within its genome were highly similar to those of PMEN1. The regions of the PMEN1 genome that differed from the ancestor contained genes associated with antibiotic resistance, transmission and virulence. We also revealed that PMEN1 was uniquely promiscuous with its DNA, donating penicillin-resistance genes and sometimes many other genes associated with antibiotic resistance, virulence and cell adherence to many genotypically diverse pneumococci. In particular, we describe two strains in which up to 10% of the PMEN1 genome was acquired in multiple fragments, some as long as 32 kb, distributed around the recipient genomes. This type of directional genetic promiscuity from a single clone to numerous unrelated clones has, to our knowledge, never before been described.

**Conclusions:**

These findings suggest that PMEN1 is a paradigm of genetic success both through its epidemiology and promiscuity. These findings also challenge the existing views about horizontal gene transfer among pneumococci.

## Background

Worldwide, over 1.6 million deaths annually are attributed to the bacterial pathogen *Streptococcus pneumoniae*, the 'pneumococcus' [1]. This bacterium is a leading cause of otitis media, sinusitis, pneumonia and meningitis, and is associated with high mortality rates in the developing world [1,2]. While many pneumococcal diseases can be successfully treated with antibiotics such as penicillin (and other β-lactams), resistance is a major global problem. The primary pneumococcal penicillin resistance determinants are three of the six penicillin-binding protein (*pbp*) genes, *pbp2x*, *pbp1a *and *pbp2b*. In penicillin-nonsusceptible pneumococci (PNSP) these genes contain mosaic sequence blocks differing from those of penicillin-susceptible pneumococci (PSP) by up to 25% nucleotide divergence [3,4]. It is believed that these mosaic blocks were acquired from closely related viridans streptococci via homologous recombination [3,5].

PNSP were first reported in the late 1960s (in the USA and Australia) [6,7]. In the late 1970s the first high-level penicillin-resistant pneumococci were reported in South Africa [8,9]. Throughout the 1980s and 1990s global penicillin nonsusceptibility rates rocketed, surpassing 50% in some regions [10]. The selective pressure of antibiotic use was enormous at this time, as antibiotics were widely used, even overused, in many countries. This selective pressure meant that pneumococci with some level of resistance to penicillin or other antibiotics had a distinct advantage over susceptible strains, which may have indirectly contributed to a change in the distribution of pneumococcal capsular types (or serotypes, defined by the antigenic polysaccharide capsule surrounding the cell) [11]. Following the introduction of a 7-valent pneumococcal conjugate vaccine (PCV7) in the year 2000 in the USA, there was a substantial reduction in PNSP levels [12]. Use of PCV7 in children provides efficacious protection against 7 of the 94 known pneumococcal serotypes [1], including those that were most commonly associated with penicillin resistance. Pneumococcal disease caused by PCV7 type pneumococci has significantly declined in countries that have introduced PCV7. Subsequently, there has been an increase in pneumococcal disease due to non-PCV7 type pneumococci [13], many of which are now also penicillin nonsusceptible [14]. 10- and 13-valent pneumococcal vaccines, containing additional serotypes, have recently been introduced, and continued surveillance over the next few years will be essential to determine their effects.

Alongside the increase of PNSP there has been an emergence of predominant and widely distributed pneumococcal clones; 43 important disease-causing clones, many of which are multidrug-resistant, have been identified by the Pneumococcal Molecular Epidemiology Network (PMEN) [15]. The first of these clones was Spain^23F^-1, here called PMEN1. This multidrug-resistant pneumococcus predominantly circulates as a vaccine serotype 23F, multilocus sequence type 81 clone (ST81^23F^); however, it has also been associated with several other serotypes, including both vaccine types and non-vaccine types [16,17]. The first PMEN1 representative was isolated in Spain in 1984 and shortly thereafter in the USA [18], the United Kingdom [16], South Africa [19,20], Hungary [21], and South America [22]. By the late 1990s it was estimated that approximately 40% of PNSP circulating in the USA were members of this clone [23] and it continues to circulate among pneumococcal populations today.

A recent study of approximately 240 pneumococci representing PMEN1 sequence type (ST)81 and related clonal variants (all belonging to clonal complex (CC) 81) showed that there is a considerable amount of genetic diversity within this lineage. This diversity, which largely results from hundreds of recombination events, indicates rapid genomic evolution and presumably allowed rapid response to selective pressures such as those imposed by vaccine and antibiotic use [17].

Here we studied a large (*n *= 426), genetically diverse, historical and global pneumococcal isolate collection with the aim to better understand the evolution of penicillin resistance within this species. We uncovered the important contribution of the PMEN1 clone to the emergence and spread of penicillin-resistant pneumococci and revealed its genetically highly promiscuous nature. Furthermore, we have been able to confidently identify the ancestor of this globally and historically important clone as being closely related to one of the oldest known PNSP.

## Results

### PMEN1 *pbp *promiscuity

Pneumococcal study isolates (Table 1) were assigned to CCs based on multilocus sequence type (MLST) data. Isolates within CCs are considered to have shared ancestry, while those in different CCs are more distantly related. Within our collection, identical or highly similar, 'nonsusceptible' *pbp *alleles and/or sequence regions were identified repeatedly among pneumococci belonging to different CCs. These *pbp *sequences were those associated with the PMEN1 reference strain (GenBank accession: NC_011900.1, hereafter simply called 'PMEN1').

**Table 1:** Isolates included in this study

Number of isolates^a^	Years	Country (n)	Source	Reference(s)
211 (174)	1937-1996	Denmark (139), USA (48), Germany (6), France (2), India (2), Malaysia (2), Singapore (2), Australia (1), Belgium (1), The Gambia (1), The Netherlands (1), Norway (1), Scotland (1), Spain (1), Sweden (1), Unknown (2)	LML	[56]
43 (43)	1948-1998	The Netherlands (6), USA (6), Portugal (5), South Africa (3), Spain (3), Czech Republic (2), Greece (2), Poland (2), Taiwan (2), Canada (1), Columbia (1), Denmark (1), England (1), Finland (1), France (1), Hungary (1), Sweden (1), Unknown (4)	LM	[15]
36 (36)	1979-1989	South Africa (36)	KPK, AVG	-
27 (27)	1969-1994	South Africa (17), Germany (8), Papua New Guinea (2)	RH	[57,58]
27 (27)	1981-1991	USA (26), South Africa (1)	LM	[59]
26 (26)	1977-1978	South Africa (26)	MRJ	[8,9,60]
22 (22)	1999-2007^b^	USA (22)	LM, BWB	-
17 (17)	1993-2005	Iceland (17)	KGK	-
9 (9)	1987-1989	Spain (9)	JL	[61-65]
8 (8)	1998-2002	USA (8)	ABB	-

Total number of isolates = 426 (excluding isolates for which genomic data were retrieved from GenBank; see Materials and methods). ^a^Number of isolates selected for *pbp *nucleotide sequencing (total *n *= 389). ^b^The year of one isolate was unknown. Source abbreviations: LML, LM Lambertsen; LM, L McGee; KPK, KP Klugman; AVG, A von Gottberg; RH, R Hakenbeck; MRJ, MR Jacobs; BWB, BW Beall; KGK, KG Kristinsson; JL, J Liñares; ABB, AB Brueggemann.

**Figure 1:**
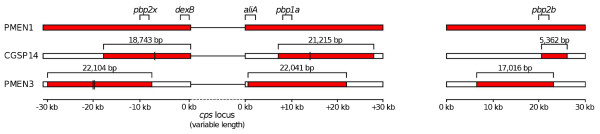
***pbp *loci and flanking regions of the PMEN1 reference, CGSP14 and PMEN3 reference genomes**. Red blocks indicate identical nucleotide sequence with substitutions or single insertions marked by black lines. The relative positions of the *pbp2x*, *pbp1a*, and *pbp2b *loci are shown, as are the two loci (*dexB *and *aliA*) flanking the capsular (*cps*) locus.

Two isolates, CGSP14 (GenBank accession NC_010582.1) and the PMEN3 reference strain (GenBank accession ABGE00000000.1, hereafter called 'PMEN3'), representing independent CCs (Table 2), shared identical *pbp2x*, *pbp1a *and *pbp2b *alleles with PMEN1. Analysis of the genomic regions flanking these *pbp *genes showed that, in fact, much larger regions of the CGSP14 and PMEN3 genomes (5,362 to 22,104 bp; Figure 1) were identical or highly similar to that of PMEN1, suggesting these regions, which conferred penicillin nonsusceptibility, were acquired from a PMEN1 representative(s) in both cases. The PMEN2 reference strain, representing another CC (Table 2), also shared identical regions (225 to 1,034 bp in length) of all three of its *pbp *alleles with PMEN1 (the intervening regions of sequence were different). A total of 12 distinct CCs were represented by isolates sharing identical *pbp *sequences in whole or part (119 to 1,376 bp) in one or two of their *pbp *alleles to PMEN1, suggesting that they too were acquired from a PMEN1 representative. Nine additional CCs were represented by isolates sharing *pbp2x *and/or *pbp2b *regions (199 to 1,436 bp) that differed from those of PMEN1 by only one or two nucleotide substitutions, again suggesting possible acquisition from a PMEN1 representative (see Additional file 1 for *pbp *allele and CC information). No other *pbp *alleles were as widely distributed as those of PMEN1.

**Table 2:** Pneumococcal isolates discussed in the text

Isolate^a^	Other name(s)	Year	Serotype	Sequence type	Clonal complex	Penicillin susceptibility^b^
PMEN1	ATCC 700669	1984	23F	81	81	I
CGSP14		2004-2005	14	15	15	R
14/5		1967	14	15	15	S
PMEN9	ATCC 700676	1993	14	9	15	S
ICE13		1998	14	9	15	S
ICE570		2002	14	9	15	S
PMEN3	ATCC 700671; SP195	1993	9V	156	156/162	I
9A/1		1962	9A	312	156/162	S
9V/5		1991	9V	162	156/162	S
9V/6		1994	9V	162	156/162	S
PMEN2	ATCC 700670	1988	6B	90	90	I
23F/4		1967	23F	7184	Singleton7184	I
PMEN18	ATCC BAA-340	1997	14	67	66	R
CDC3059-06		2005	19A	199	199	I
CDC0288-04		Unknown	12F	220	218	S
USA18		1999	12F	218	218	S
TIGR4		1991	4	205	205	Unk

^a^PMEN refers to Pneumococcal Molecular Epidemiology Network reference strains. ^b^S = MIC ≤ 0.06 μg/ml; I = MIC 0.12 to 1 μg/ml; R = MIC ≥ 2.0 μg/ml; Unk = unknown.

### Extensive PMEN1 genomic recombination

**Figure 2:**
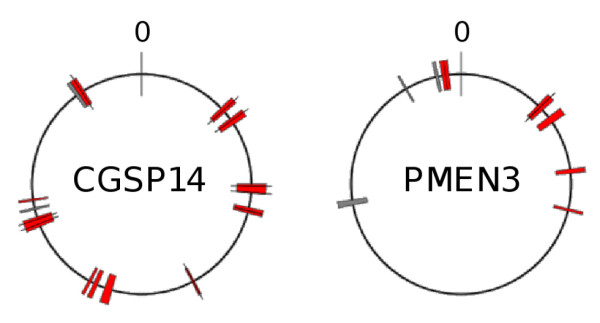
**PMEN1 reference genome regions putatively acquired by CGSP14 and the PMEN3 reference**. Putative recombination regions marked by colored blocks and mapped to PMEN1 reference genome coordinates. Red blocks: regions identical or highly similar to that of the PMEN1 reference only. Gray blocks: regions identical or highly similar to those of the PMEN1 reference and one or more CC66 representative(s). Single nucleotide substitutions or insertions are marked with black lines. Origin of replication marked as '0'.

Given that the CGSP14 and PMEN3 genomes each contained three large, independent regions that were putatively acquired from PMEN1, we used whole genome sequence data generated on the Illumina platform to assess whether other regions of the PMEN1 genome were also shared by CGSP14 and PMEN3 (both of which represent multidrug-resistant clones, the latter of which is also globally distributed). We found that, in fact, 15 regions (1,663 to 32,312 bp, totalling 211,963 bp, approximately 9.5% of the genome) of the CGSP14 chromosome were identical to those of PMEN1 or differed by only 1 to 2 nucleotides across a minimum of 7,537 bp (Figure 2; Additional file 2), and were not identical in any of four CGSP14 ancestral representative genomes. The ancestral representatives (14/5, the PMEN9 reference strain, ICE13 and ICE570; Table 2) were chosen from our collection because they represented older and/or penicillin-susceptible members of the CGSP14 CC and so likely resembled the true ancestor of the penicillin-resistant CGSP14. Importantly, this allowed us to identify and exclude identical sequence that was shared by descent, whereas regions of the CGSP14 genome that differed from its ancestral representatives but were identical to those of PMEN1 were most likely acquired from PMEN1 through the process of recombination. The ancestral isolates were generally all identical or highly similar to each other at the genomic regions in question; sequences from these isolates were also highly similar to CGSP14 in the regions flanking the putative recombination imports, confirming their suitability as ancestral representatives.

Following a similar comparison of PMEN3 and its ancestral representatives (9A/1, 9V/5 and 9V/6; Table 2), we identified nine PMEN3 genomic regions (4,662 to 22,104 bp, totalling 117,689 bp, approximately 5.3% of the genome) that were identical to sequences from PMEN1 or differed by only two nucleotides across 22,104 bp, and were different from those of the ancestral representatives (Figure 2; Additional file 2). We suggest that these regions were also acquired by PMEN3 from PMEN1 through the process of recombination.

The CGSP14 and PMEN3 putative recombinogenic regions contained 213 and 108 whole or part coding sequences, respectively (Additional file 2). In addition to the penicillin resistance-conferring *pbp2x*, *pbp1a *and *pbp2b *genes, the following coding sequences associated with enhanced virulence or colonization potential were included: the CGSP14 *bgaA*, *clpX*, SPN23F_05810 and SPN23F_20890 genes associated with enhanced host colonization ability [24]; the CGSP14 virulence-associated *cbpJ*, *msmK*, chloramphenicol acetyltransferase and zeta toxin/epsilon antitoxin genes [25-27] (the latter two of which are situated on the CGSP14 conjugative transposon); and the PMEN3 virulence-associated *cbpD*, *cbpJ *and *clpC *genes [26].

The possibility that the putative imported regions were acquired by CGSP14 and PMEN3 from non-PMEN1 representative pneumococci cannot be ignored. However, the majority, 13 and 6, respectively, of regions described here were not identical among any of 96 additional pneumococcal genomes, representing a diverse range of 31 independent CCs (listed in Additional file 3, excluding 23F/4 and CC66 isolates). Two and three of the regions, respectively (Figure 2), were identical or highly similar among isolates representing CC66, and so it is possible that those regions may have been acquired from a CC66 representative (also see Additional file 4). Although our collection cannot capture the full complement of pneumococcal genetic diversity, given these results and the extremely high similarity of the putative import regions to the PMEN1 genome, we believe that they originated from a PMEN1 representative. Thus, these data suggest that DNA originating from the PMEN1 clone has greatly influenced the genomic evolution of at least two unrelated pneumococcal clones.

### Ancestors of PMEN1

The Genome Comparator tool [28] was used to screen 104 pneumococcal genomes, representing a very diverse range of CCs and serotypes (Additional file 3), for regions of DNA (aside from the *pbps*) that were identical to PMEN1. We identified 1,990 coding sequences from the PMEN1 GenBank Accession NC_011900.1 file (totaling 1,833,672 bp, approximately 83% of the genome). Comparable coding sequences in the query genomes were identified by BLASTn search if they shared ≥ 70% nucleotide sequence identity and could be aligned at 100% length. Consequently, coding sequences spanning the ends of assembly contigs could not be identified.

**Figure 3:**
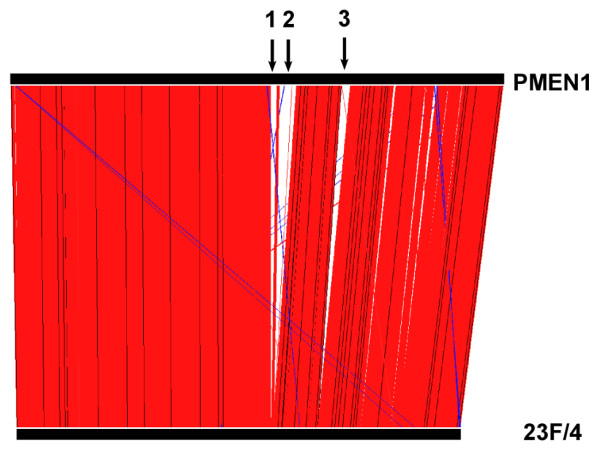
**Artemis Comparison Tool comparison of the PMEN1 reference and 23F/4 genomes**. Red lines indicate homologous nucleotide sequence matches. Blue lines indicate reverse complement nucleotide sequence matches. Black arrows indicate large regions present in the PMEN1 reference genome but absent from the 23F/4 genome: (1) Na^+^-dependent ATPase island; (2) ICE*Sp*23FST81 element; and (3) ɸMMI phage.

Surprisingly and uniquely, the analyses showed that 23F/4, the first PNSP isolated from Australia (Table 2), was very likely to be an ancestral representative of PMEN1, since approximately 95% of the identified coding sequences were ≥ 98% similar to those of PMEN1. Approximately 75% of identified coding sequences in 23F/4 were identical to PMEN1 and over one-third of the coding sequences that were not identical differed by only one or two nucleotide substitutions. The *pbp2x*, *pbp1a*, and *pbp2b *genes and three of the seven MLST loci were not identical. Additionally, a comparison of the PMEN1 and 23F/4 genomes, performed using the Artemis Comparison Tool [29] using all available sequence (both coding and non-coding sequence; Figure 3), clearly showed three large regions of the PMEN1 genome that were partially or completely missing from the 23F/4 genome and were presumably acquired by a more recent ancestor of PMEN1. These were the ICE*Sp*23FST81 element, the ɸMMI phage and a Na^+^-dependent ATPase island, all associated with increased colonization and virulence potential [30-33].

**Figure 4:**
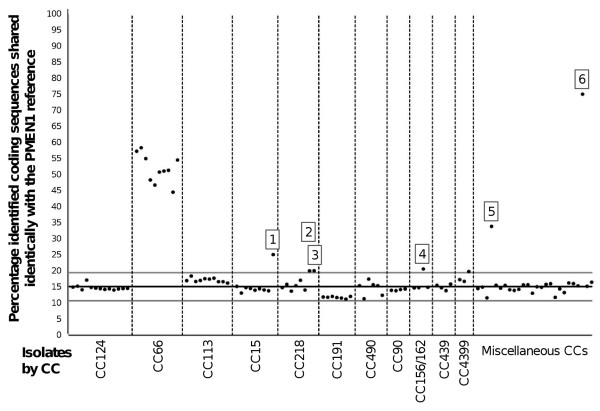
**Percentage PMEN1 reference coding sequences identified identically within 104 pneumococcal genomes**. Points represent independent pneumococcal isolates. Isolates representing CCs for which *n *≥ 3 are labeled by CC. Isolates representing CCs for which *n *< 3 are labeled as 'Miscellaneous CCs'. Black line represents mean percentage identical coding sequences ± 2 standard deviations (gray lines). Isolates of interest are labeled: (1) CGSP14; (2) USA18; (3) CDC2088-04; (4) PMEN3; (5) CDC3059-06; and (6) 23F/4 (also see Additional file 4).

The Genome Comparator analyses also revealed that the majority (87, 83.6%) of genomes analyzed - representing 31 unique CCs and 30 serotypes - shared 15.1% ± 2 standard deviations (excluding 12 outliers with > 30% identical coding sequences) of the identified coding sequences identically with PMEN1 (Figure 4). We suggest that these 'baseline' coding sequences were shared through ancestral descent, that is, they were identical in the most recent common ancestor of these clones and have not undergone subsequent change. (For purposes of comparison, the same analysis was performed using pneumococcal genome TIGR4 (GenBank accession AE005672.3) as the reference and a similar baseline mean level of identical sequence of 14.5% was observed (Additional file 5).

Isolates belonging to CC66 shared between 44.5% and 58.4% coding sequences identically with PMEN1. These isolates included PSP and were dated from 1952, preceding the estimated origin of PMEN1. Since the proportion of identical coding sequences between CC66 and PMEN1 was much greater than between those of PMEN1 and the other CCs represented here, we conclude that PMEN1 was ancestrally more closely related to CC66 than to other CCs, which supports the findings of Donati and colleagues [34] (also see Additional file 4).

## Discussion

To our knowledge, our collection represents the largest historical pneumococcal collection ever compiled and includes the greatest number of early (1960s to 1980s) PNSP in a single study. This has provided us with a unique opportunity to study pneumococcal evolution. Within our diverse isolate collection, whole or part *pbp *alleles that were identical or highly similar to those of the PMEN1 reference strain were identified from pneumococci representing a total of 24 distinct CCs. Two isolates, CGSP14 and PMEN3, shared *pbp2x*, *pbp1a *and *pbp2b *sequences identically with PMEN1, and shared highly similar sequences in the *pbp *flanking regions. The full extent of sharing all three of these PMEN1 *pbp *sequences has never before been uncovered (nor have the large regions around all three *pbps*), although previous authors have independently noted *pbp *similarities between CGSP14, PMEN3, PMEN2 and PMEN1 [16,35-38]. Highly similar *pbp2x *and *pbp2b *nucleotide sequences have also been independently identified among numerous pneumococcal and viridans streptococcal clones [37,39,40]. Shared resistance-conferring *pbp *sequences other than those associated with PMEN1 have also been described previously [5,41,42] and were identified among our isolate collection, though at much lower frequency (data not shown). Additionally, data from other authors have suggested that fluoroquinolone resistance determinants have been donated from PMEN1 to numerous unrelated pneumococcal clones [43].

It is very unlikely that identical or highly similar sequences were assembled independently in different CCs, but rather that they have been transferred, as whole or part alleles, between unrelated pneumococcal clones at least once for each independent CC. Note that the establishment of defined CCs must have occurred prior to acquisition of these *pbp *alleles, since we also identified penicillin-susceptible members of these clones (that is, CC15^14^, CC156/162^9A/V^).

The predominance of the PMEN1-like *pbp *sequences within our isolate collection suggests that the PMEN1 reference *pbp2x*, *pbp1a and pbp2b *sequence combination may provide an evolutionary selective advantage over other resistance-conferring *pbp *alleles, which is consistent with recent experimental data suggesting certain combinations of *pbp2x *and *pbp1a *alleles are more advantageous than others [44]. A study of genomic evolution within the PMEN1 lineage also provided evidence consistent with the hypothesis that the *pbp2x *and *pbp1a *alleles were positively selected [17]. *pbp2x *is upstream and *pbp1a *is downstream of the capsular (*cps*) locus in the pneumococcal genome. The authors noted that the boundaries of recombination events affecting the *cps *locus appeared to be restricted by the nearby *pbp2x *and *pbp1a *loci. It is known that both of these loci and the *cps *locus can be transferred simultaneously between pneumococci [45], and thus the restriction within the PMEN1 lineage is likely not entirely mechanistic.

In addition to the *pbp *loci and flanking regions, CGSP14 and PMEN3 acquired multiple genomic regions, totaling approximately 9.5% and approximately 5.3% of the genome, respectively, from PMEN1 representative(s). This is consistent with two recent studies that demonstrated that multiple fragment recombination had taken place *in vivo*, between pneumococci isolated from a single patient [46] and among vaccine escape strains isolated in the USA [47]. The suggestion that a significant proportion of the genome may be acquired from a single pneumococcal donor was recently supported mechanistically by Attaeich and colleagues [48]. These authors showed that cellular levels of pneumococcal SsbB protein are sufficient to maintain an intracellular pool of internalized DNA of up to 1.15 Mb of length (approximately 50% of the genome). Such a pool of genetic material, potentially originating from a single donor cell, could then be used for successive rounds of recombination.

We cannot know for certain whether the CGSP14 and PMEN3 imported regions were acquired simultaneously in a single event or via successive recombination events. Either way, the data strongly suggest that they were acquired from a PMEN1 representative. It should also be noted that recombination events additional to those described here, and involving acquisition of DNA from other pneumococcal clones or other species, may also have altered the CGSP14 and PMEN3 genomes.

The genomic regions acquired from the PMEN1 donor may also have contributed to the virulence and host colonization ability of the recipients. CGSP14 was multidrug-resistant and isolated from a child suffering from necrotizing pneumonia complicated with hemolytic uremic syndrome [35]. PMEN3 represents another globally distributed multidrug-resistant clone [15]. The putative recombinogenic regions acquired by both of these pneumococci included several genes associated with virulence or host colonization potential. Interestingly, large sections of the CGSP14 transposon, which was shown previously to contain regions of similar sequence to those of the PMEN1 ICE*Sp*23FST81 element [35], were in fact identical to those of PMEN1. Two interpretations of the regions of similarity within the ICE*Sp*23FST81 element are possible. One is convergent evolution between PMEN1 and CGSP14, with both strains independently acquiring similar, recently diverged elements. Alternatively, a more divergent element may have been acquired by CGSP14 that was subsequently modified through recombination with DNA from PMEN1. The mosaic nature of these variable conjugative elements [17] makes distinguishing these two hypotheses difficult.

Finally, we showed that one of the first reported PNSP (23F/4) represented the ancestor of PMEN1. The year of isolation of 23F/4 (1967) correlated with the predicted date of emergence of the PMEN1 lineage (approximately 1970), although the country of isolation (Australia) did not match the predicted region of PMEN1 origin (Europe) [17]. Despite its high overall genomic similarity to PMEN1, the 23F/4 ST, which differed from that of PMEN1, has not been identified amongst modern pneumococci. Perhaps the differences between these two genomes could explain the differences in disseminative success of these two STs. The acquisition of more favorable *pbp2x*, *pbp1a *and *pbp2b *alleles in the PMEN1 genome provides a partial explanation; however, other changes and/or acquisition of other loci have also likely played an important role. The PMEN1 ICE*Sp*23FST81 element, Na^+^-dependent ATPase island and ɸMMI phage were absent from the 23F/4 genome. The ICE*Sp*23FST81 element carries both tetracycline and chloramphenicol resistance determinants [30] plus a *umuDC*-like gene shown to provide protection against UV damage [32]. The Na^+^-dependent ATPase island and a ɸMMI phage closely related to that of PMEN1 have been associated with an increased incidence of invasive pneumococcal disease [33] and increased cell adherence capabilities [31], respectively.

Any or all of these regions may have provided PMEN1 with a selective advantage, allowing it to persist among the greater pneumococcal population for the past three decades. Alternatively, the combination of these regions with the PMEN1 reference *pbp *genes and the 23F/4 genetic background may have been the most important factor. In any case, the subsequent global dissemination of PMEN1 likely aided the spread of its *pbp *alleles and other regions of its genome to numerous unrelated pneumococci. Some of the recipient pneumococci might subsequently have transferred these PMEN1-like sequences to additional pneumococcal clones.

Recently, through comparison of multiple representatives within the PMEN1 CC, Croucher *et al. *[17] showed that the PMEN1 lineage contains a vast amount of genetic diversity, largely resulting from horizontal acquisition of DNA via recombination. Through comparison of genomic sequences across a genetically diverse collection of pneumococci, our study has greatly increased our understanding of the emergence of the PMEN1 clone and its subsequent contribution to the genomic evolution and spread of penicillin resistance determinants among other pneumococci. It is well understood that many bacterial species, particularly the pneumococcus, frequently undergo horizontal gene transfer; however, the directional genetic promiscuity described here, from a single epidemiologically successful clone to numerous unrelated clones, has not been demonstrated before in pneumococci, or other bacterial species as far as we are aware.

Antibiotics are among the most influential global public health successes, but impose selective pressures that drive bacterial genomic evolution. PMEN1 is an excellent example of a bacterium that has become resistant to multiple antibiotics and that has evolved to become very successful in colonization, transmission, and causing disease. Moreover, PMEN1 has subsequently shared its advantageous DNA with other unrelated pneumococci. Studies such as this one may help to identify ways to counteract these biological changes and preserve the value of antibiotics for the future.

## Materials and methods

### Strain collection

A unique collection of 426 global PSP and PNSP dated 1937 to 2007 was studied, as summarized in Table 1. Isolates included one of the first PNSP reported from Australia [6], the first high-level PNSP reported from South Africa [8,9] and the 43 PMEN reference strains [15]. The Statens Serum Institut, Denmark provided 211 pneumococci dated 1937 to 1996. These isolates included both PSP (*n *= 203), PNSP (*n *= 7) and an isolate of unknown penicillin susceptibility (*n *= 1). Following the analyses of MLST and *pbp *sequence data from these isolates, the 43 PMEN clones (including both PSP (*n *= 18) and PNSP (*n *= 25)) and additional modern (1990s to 2000s) PSP (*n *= 38) and PNSP (*n *= 9) that had been recently genotyped by A Brueggemann/L McGee and represented CCs of interest, were selected for inclusion. In addition, a literature search was performed to identify studies that reported early PNSP. The authors of those papers were contacted and isolates were requested; the majority of isolates were still available and were added to our collection. The available isolates comprised PNSP dated 1969 and 1972 from Papua New Guinea (*n *= 2), PSP (*n *= 8) and PNSP (*n *= 71) dated 1977 to 1989 from South Africa, PSP (*n *= 1) and PNSP (*n *= 26) dated 1981 to 1991 from the USA, PNSP dated 1987 to 1989 (*n *= 9) from Spain and PNSP dated 1986 to 1994 from Germany (*n *= 8). Finally, genomic sequence data for 16 isolates were retrieved from the GenBank database. These isolates included both PSP (*n *= 5), PNSP (*n *= 4) and seven pneumococci of unknown penicillin susceptibility. Serotype and penicillin susceptibility data are summarized by decade in Additional file 6. Where not previously published, isolates were serotyped by Quellung reaction and penicillin minimum inhibitory concentrations were obtained by Etest (bioMérieux Basingstoke, United Kingdom) as per the manufacturer's guidelines.

### DNA extraction

Single pneumococcal colonies were cultured on Columbia agar with sheep blood (Oxoid Basingstoke, United Kingdom) and incubated overnight at 37°C + 5% CO_2_. Three to four sweeps of growth were subcultured onto tryptic soy agar (Oxoid) with 1,000 U ml^-1 ^catalase (Oxoid) and spread with a swab before further overnight incubation at 37°C and 5% CO_2 _(at Oxford); or cultured in serum broth for 7 h (at the Statens Serum Institut). Pneumococcal growth was suspended in 1 ml phosphate buffered saline (pH 7.4; Fisher Bioreagents Loughborough, United Kingdom) and centrifuged at 7,600 rpm for 10 minutes. Extractions were completed using the DNeasy kit (Qiagen, West Sussex, United Kingdom) as per the manufacturer's instructions.

### MLST and clonal complex designation

Where not previously published (*n *= 352) multilocus sequence types were determined for all isolates [49]. MLST data for all of our isolates and those deposited in the pneumococcal MLST database [50] were analyzed by the goeBURST [51] method to predict CC group and sub-group founders. Isolates were assigned to CCs so that only single/double locus variants of the group founder and any additional single locus variants of large (n ≥ 5 STs) sub-group founders were included. CCs were named according to the predicted group founder(s) ST(s). When goeBURST could not distinguish a group founder the group was designated NoneX, where X was the ST of lowest numerical value in the group. When isolates could not be assigned to a CC due to a lack of closely related STs, they were designated SingletonX, where X was the isolate ST.

### *pbp *profiling

*pbp2x *(1,673 bp), *pbp1a *(901 bp) and *pbp2b *(1,272 bp) nucleotide sequences for 389 selected isolates were retrieved from GenBank (*n *= 36 sequences) or determined by conventional PCR and Sanger sequencing (*n *= 1,131 sequences). We chose 174 of 211 isolates from the Statens Serum Institut for *pbp *nucleotide sequencing because they represented a wide range of serotypes, STs and isolation dates. All isolates from each of the other sources were also included. PCR reactions containing 1 μl DNA extract, 13.5 μl Hotstar Taq DNA Polymerase master mix (Qiagen, West Sussex, United Kingdom), 0.5 μl each of the forward and reverse primers (100 μM) (Additional file 7) were prepared to a final volume of 25 μl. Cycling parameters were as follows: initial denaturation of 95°C for 180 s, followed by 35 (40 for *pbp2x*) cycles of 95°C for 30 s, 55°C (58°C for *pbp1a*) for 30 s and 72°C for 60 s followed by a final extension period of 72°C for 10 minutes. Products were precipitated by 20% polyethylene glycolate/2.5 M NaCl, washed in 70% ethanol and rehydrated in 10 to 20 μl nuclease-free H_2_O (Sigma Gillingham, United Kingdom). Sequencing reactions containing 2 μl rehydrated PCR product, 1.75 μl 5x sequencing buffer, 4 μl primer (1 μM) (Additional file 7) and 0.5 μl BigDye^® ^(Applied Biosystems, Paisley, United Kingdom) were prepared in a final volume of 10 μl. Cycling parameters were as follows: 25 cycles of 96°C for 10 s, 50°C for 5 s and 60°C for 2 minutes. Sequencing products were precipitated with 5 M sodium acetate (Sigma) solution, washed with 70% ethanol and subsequently separated and detected with an ABI Prism 3730 automated DNA sequencer (Applied Biosystems, Paisley, United Kingdom). Sequences were aligned by MUSCLE [52] and imported to MEGA5 [53] for visual comparison. Unique *pbp *sequences were assigned unique allele numbers.

### Whole-genome sequencing

We selected 96 isolates for whole genome sequencing (Additional file 3) using the following criteria. First, isolates that were not closely related to PMEN1 based on MLST data but shared identical *pbp *alleles or partial *pbp *alleles to those of the PMEN1-reference strain. The selected isolates were distributed through time, by geographic location and by genotype in order to capture the greatest possible amount of genetic diversity. Second, isolates representing CCs of interest (for example, major international CCs) for which both historical and modern isolate representatives were available. Inclusion of the historical representatives was crucial, as it allowed us to differentiate identical/highly similar sequence imports obtained exogenously versus identical sequence due to common ancestry. Within these CCs the selected isolates were distributed through time, by geographic location, by serotype and by *pbp *allele combination, in order to capture the greatest possible amount of genetic diversity.

DNA was extracted to a final concentration of 20 ng/μl as described above. Sequencing was via the Illumina platform as previously described [17]. As per standard protocols, multiplex library construction with a 200 bp insertion size was followed by 54 nucleotide paired-end sequencing. Illumina reads were assembled to contigs using the Velvet software [54]. Data for seven genomes were rejected based on technical problems. K-mer lengths were optimized to the maximum length achieving an average of ≥ 20× read coverage across assemblies. Resulting contigs were between 41 bp and 328,742 bp length (mean = 3,797 bp; see Additional file 4 for further details). Contigs were deposited in a BIGSdb database [28] and in the European Nucleotide Archive (see Additional file 8 for accession numbers). Contigs were ordered to the PMEN1 reference genome using ABACAS [55] and viewed with the Artemis Comparison Tool [29], allowing for the extraction of selected sequence regions spanning > 1 contig.

### Identification of recombination fragments from PMEN1

The BIGSdb Genome Comparator tool, which utilizes the BLAST algorithm, was used to compare PMEN1 coding sequences to those of CGSP14, PMEN3 and their respective predicted ancestral representatives (Table 2). Comparable coding sequences in the query genomes were identified by BLASTn search if they shared ≥ 70% nucleotide sequence identity (to allow for the identification of variable sequences among conserved protein classes [28] and to avoid bias towards sequence variants specific to PMEN1) and could be aligned at 100% length (to ensure that any coding sequences identified as identical to PMEN1 were indeed identical across their entire length). Potential recombination regions were indicated by coding sequences shared identically by PMEN1 and CGSP14 or PMEN3, but not by any of the respective ancestral representatives. Sequence regions spanning the coding sequences of interest plus 5 kb flanking either side were extracted from the PMEN1, CGSP14, PMEN3 and ancestral representative genomes, aligned and imported into MEGA5 [53] to produce variable site maps for visual inspection (Additional file 9). Recombination regions were classified as the maximum length of sequence over which CGSP14 or PMEN3 were identical to PMEN1 but different from their ancestral representatives.

### Identification of PMEN1 ancestral representatives

Genome comparator was used to screen 104 isolates (Additional file 3) representing 34 CCs and 31 serotypes for coding sequences identical to those of the PMEN1 reference (parameters as above). 23F/4 was identified as sharing a high percentage of coding sequences identically with PMEN1 and was thus subjected to further analysis. PMEN1 coding sequences that differed in 23F/4 were extracted from the PMEN1 whole-genome sequence and BLASTed against the 23F/4 Velvet contigs in the BIGSdb. Sequences were aligned and percentage nucleotide difference was calculated. 23F/4 contigs were ordered against the PMEN1 chromosome by ABACAS [55] and viewed with the Artemis Comparison Tool [29] to identify genomic regions present in PMEN1 but absent from 23F/4.

## Abbreviations

bp: base pair; CC: clonal complex; *cps*: capsular; Mb: mega base pair; MLST: multilocus sequence typing; *pbp*: penicillin-binding protein; PCR: polymerase chain reaction; PCV7: 7-valent pneumococcal conjugate vaccine; PMEN: Pneumococcal Molecular Epidemiology Network; PNSP: penicillin-nonsusceptible pneumococci; PSP: penicillin-susceptible pneumococci; ST: sequence type.

## Competing interests

The authors declare that they have no competing interests.

## Authors' contributions

KLW and ABB designed the study, analyzed data, wrote the paper; LML, LM, AVG, JL, MRJ, KGK, BWB, KPK, RH and ABB provided bacterial isolates; KLW, LM, JL, BWB, RH and ABB generated *pbp *and/or MLST sequence data; LML, AVG, JL, KGK, BWB, KPK and RH provided serotype data; KLW, LML, LM, AVG and RH performed susceptibility testing; KLW and LML extracted genomic DNA for whole-genome sequencing; JP and SDP provided whole genome sequencing data; KLW and NJC assembled whole genome sequence data. All authors discussed the results, read and approved the manuscript for publication.

## Supplementary Material

Additional file 1 **Tables S1 to S3**. Lists of the *pbp *allele sequence regions that were identical or highly similar to those of the PMEN1 reference strain. Click here for file

Additional file 2 **Tables S4 and S5**. Details of the regions of the CGSP14 and PMEN3 genomes, respectively, that were identified as putatively recombinogenic and acquired from PMEN1. Click here for file

Additional file 3 **Table S6**. More information (for example, serotype and penicillin susceptibility) about the isolates for which whole-genome sequences were analyzed in this study. Click here for file

Additional file 4 **Supporting Results and Methods**. More information about MLST and *pbp *diversity, the imported regions of CGSP14 and PMEN3, coding sequences found in both PMEN1 and isolates of CC66, outlier isolates highlighted in Figure 4 and methodological details related to whole-genome sequence assemblies. Click here for file

Additional file 5 **Figure S2**. Depiction of the results of a Genome Comparator analysis of 104 pneumococcal genomes compared to the TIGR4 reference genome. Click here for file

Additional file 6 **Table S7**. Summary of the 442 isolates included in this study, stratified by era of isolation. Click here for file

Additional file 7 **Table S8**. Details of the primers used for PCR and conventional sequencing of regions of *pbp2x*, *pbp1a *and *pbp2b*. Click here for file

Additional file 8 **Table S9**. Details of the metadata for the 442 isolates included in this study. Click here for file

Additional file 9 **Figure S3**. A variable site map of one PMEN3 putative imported region and flanking regions. Click here for file
