# Supplementary material for: The multidrug-resistant PMEN1 pneumococcus is a paradigm for genetic success
Source: Genome Biol. 2012 Nov 16;13(11):R103. doi: 10.1186/gb-2012-13-11-r103 (PMC3580495; doi:10.1186/gb-2012-13-11-r103)
Supplement: Additional file 1 — Tables S1 to S3. Lists of the pbp allele sequence regions that were identical or highly similar to those of the PMEN1 reference strain. [file gb-2012-13-11-r103-S1.PDF]

Table S1. ***pbp2x*** **allele sequence regions identical or highly similar to those of the PMEN1 reference strain.**

Allele no.	PMEN1 Matching Regions		Nucleotide Substitution Positions (bp)	CC Representatives
	Start (bp)	End (bp)		
67	NA (PMEN1)		NA	15, 81, 156/162
75	298	End	None	18
80	298	1331	None	90
85	317	1526	None	None41, Singleton7248
88	343	End	None	90
89	310	End	None	242
86	253	End	291	271/320
107	169	1604	618	558
117	298	1331	1132	90
119	298	1604	735	22
121	298	1331	596	90
68	259	1514	735, 753	87/88
77	253	End	618, 1199	490, 2090
99	138	284	None	384
	335	End	617, 1199	
100	442	End	618, 1199	230, 304
118	439	End	1369, 1374	Singleton7256
129	169	End	618, 1199	1094
141	181	End	618, 1662	1106

Table S2. ***pbp1a*** **allele sequence regions identical or highly similar to those of the PMEN1 reference strain.**

Allele no.	PMEN1 Matching Regions		Nucleotide Substitution Positions (bp)	CC Representatives
	Start (bp)	End (bp)		
72	NA (PMEN1)		NA	15, 81, 156/162
77	62	225	None	18, 81, 156/162
	464	End	None	
78	Start	349	None	2090
79	Start	225	None	90
	464	End	None	
101	Start	225	None	384
	464	669	None	
86	71	349	222	90
	635	End	None	

Table S3. ***pbp2b*** **allele sequence regions identical or highly similar to those of the PMEN1 reference strain.**

Allele no.	PMEN1 Matching Regions		Nucleotide Substitution Positions (bp)	CC Representatives
	Start (bp)	End (bp)		
62	NA (PMEN1)		NA	15, 81, 156/162
61	Start	232	None	87/88
71	Start	232	None	Singleton175
73	Start	217	None	15, 384
106	Start	232	None	90
111	Start	232	None	1106
130	Start	232	None	1094
76	Start	232	74	2090
79	Start	232	74	490
81	Start	163	74	242
85	Start	232	74	230
86	Start	232	74	271/320
	392	514	None	
87	Start	232	74	66
	394	514	None	
95	Start	232	74	344
96	Start	232	74	558
	396	514	None	
104	Start	232	74	81
	396	514	None	
105	Start	199	74	22, 490
129	50	232	74	5431
