# Supplementary material for: The multidrug-resistant PMEN1 pneumococcus is a paradigm for genetic success
Source: Genome Biol. 2012 Nov 16;13(11):R103. doi: 10.1186/gb-2012-13-11-r103 (PMC3580495; doi:10.1186/gb-2012-13-11-r103)
Supplement: Additional file 2 — Tables S4 and S5. Details of the regions of the CGSP14 and PMEN3 genomes, respectively, that were identified as putatively recombinogenic and acquired from PMEN1. [file gb-2012-13-11-r103-S2.PDF]

Table S4. Regions of the CGSP14 genome identified as putatively recombinogenic and acquired from PMEN1.

Region	PMEN1 reference coordinates		Size (bp)	Included CDS	CDS predicted function	CDS in whole or part
	Start	End				
1	285184	303926	18743	*agaW*	N-acetylgalactosamine-specific phosphotransferase system (PTS), IIC component	Part
				agaD	N-acetylgalactosamine-specific phosphotransferase system (PTS), IID component	Whole
				SPN23F_03000	Hypothetical protein	Whole
				SPN23F_03010	Heparinase II/III-like protein	Whole
				SPN23F_03020	LacI family transcriptional regulator	Whole
				SPN23F_03030	Membrane protein	Whole
				SPN23F_03040	Hypothetical protein	Whole
				SPN23F_03050	DNA-binding protein	Whole
				mraW	S-adenosyl-methyltransferase MraW	Whole
				ftsL	Cell division protein	Whole
				pbp2x	Penicillin-binding protein 2x	Whole
				mraY	Phospho-N-acetylmuramoyl-pentapeptide-transferase	Whole (1 nucleotide substitution at position 295551)
				clpL	ATP-dependent protease ATP-binding subunit ClpL	Whole
				SPN23F_03120	Group II intron maturase	Whole
				luxS	S-ribosylhomocysteinase	Whole
				SPN23F_03140	Hypothetical protein	Whole
				dexB	Glucan 1,6-alpha-glucosidase	Whole
				wzg / *cpsA*	Capsule biosynthesis integral membrane regulatory protein Wzg	Part
2	330194	351408	21215	SPN23F_03400	Cell wall surface anchored protein	Part
				pbp1A	Penicillin-binding protein 1A	Whole
				recU	Holliday junction-specific endonuclease	Whole
				SPN23F_03430	Hypothetical protein	Whole
				SPN23F_03440	DivIVA protein	Whole
				SPN23F_03450	RNA methylase family protein	Whole
				SPN23F_03460	Hypothetical protein	Whole (1 nucleotide substitution at position 338114)
				gnd	6-phosphogluconate dehydrogenase	Whole
				csrR	Response regulator protein	Whole
				cbpJ	Choline binding protein J	Whole
				SPN23F_03500	Major facilitator superfamily protein	Whole
				mvaK1	Mevalonate kinase	Whole

				mvaD	Mevalonate diphosphate decarboxylase	Whole
				mvaK2	Phosphomevalonate kinase	Whole
				fni	Isopentenyl pyrophosphate isomerase	Whole
				SPN23F_03570	Hypothetical protein	Whole
				SPN23F_03580	Sensor histidine kinase	Whole
				SPN23F_03590	Response regulator protein	Whole
3	554668	584068	29401	*rplK*	50S ribosomal protein L11	Whole
				rplA	50S ribosomal protein L1	Whole
				SPN23F_05720	Hypothetical protein	Whole
				SPN23F_05730	Hypothetical protein	Whole
				SPN23F_05740	ABC transporter ATP-binding protein	Whole
				SPN23F_05750	Hypothetical protein	Whole
				SPN23F_05760	Hypothetical protein	Whole (1 nucleotide substitution at position 560786)
				SPN23F_05770	Hypothetical protein	Whole
				SPN23F_05780	Hypothetical protein	Whole
				SPN23F_05790	Surface-anchored serine protease	Whole
				SPN23F_05800	Sugar phosphotransferase system (PTS), IIA component	Whole
				SPN23F_05810	Sugar phosphotransferase system (PTS), IIB component	Whole
				SPN23F_05820	Sugar phosphotransferase system (PTS), galactitol-specific family, IIC component	Whole
				bgaA	Surface anchored beta-galactosidase	Whole
				SPN23F_05840	Hypothetical protein	Whole
				SPN23F_05850	Hypothetical protein	Whole
				SPN23F_05860	Hypothetical protein	Whole
				SPN23F_05890	rRNA methylase	Whole
				SPN23F_05900	Hypothetical protein	Whole (1 nucleotide insertion in CGSP14 at position 581754)
				SPN23F_05910	Sodium hydrogen exchange transporter	Whole
4	631008	639926	8919	*lctO*	L-lactate oxidase	Whole
				SPN23F_06400	Coenzyme	Whole
				thiM	Hydroxyethylthiazole kinase	Whole
				thiE	Thiamine-phosphate pyrophosphorylase	Whole
				SPN23F_06440	Hypothetical protein	Whole
				SPN23F_06450	ABC transporter ATP-binding protein	Whole
				SPN23F_06460	Hypothetical protein	Whole
				SPN23F_06470	Coenzyme	Whole
				SPN23F_06480	Hypothetical protein	Whole

				SPN23F_06490	Hydroxyethylthiazole kinase	Part
5	640607	642269	1663	*thiE*	Thiamine-phosphate pyrophosphorylase	Part
				thiD	Phosphomethylpyrimidine kinase	Whole
6	642471	649059	6589	SPN23F_06520	Regulator	Part
				SPN23F_06530	Hypothetical protein	Whole
				SPN23F_06540	Metal transporting P-type ATPase	Whole
				spxB	Pyruvate oxidase	Whole
				SPN23F_06560	Glyoxalase family protein	Whole
7	934640	942176	7537	SPN23F_09650	Hypothetical protein	Part
				SPN23F_09660	Alpha-amylase	Whole
				SPN23F_09670	Hypothetical protein	Whole
						1 nucleotide substitution at position 938232
				SPN23F_09700	Epsilon antitoxin	Whole
				SPN23F_09710	Zeta-toxin	Whole
				SPN23F_09731	Hypothetical protein	Whole
				SPN23F_09740	*Tn5252*, ORF 10 protein	Whole
				SPN23F_09750	*Tn5252*, ORF 9 protein	Part
8	1207734	1231897	24164	SPN23F_12410	Integrase	Whole
				SPN23F_12420	Hypothetical protein	Whole
				SPN23F_12430	Relaxase	Whole
				SPN23F_12440	Mobilisation protein	Whole
				SPN23F_12450	Mobilisation protein	Whole
				SPN23F_12460	Hypothetical protein	Whole
				SPN23F_12470	DNA helicase II, UvrD	Whole
				SPN23F_12480	Hypothetical protein	Whole
				SPN23F_12490	Hypothetical protein	Whole
				SPN23F_12500	Hypothetical protein	Whole
				SPN23F_12510	NTPase protein	Whole
				SPN23F_12520	Hypothetical protein	Whole
				SPN23F_12530	Phosphoserine phosphatase	Whole
				SPN23F_12540	Hypothetical protein	Whole
				SPN23F_12550	Hypothetical protein	Whole
				SPN23F_12560	Hypothetical protein	Whole
				SPN23F_12570	Hypothetical protein	Whole
				rep	Replication protein	Whole
				SPN23F_12590	Chloramphenicol acetyltransferase	Whole
				SPN23F_12600	Hypothetical protein	Whole
				SPN23F_12610	Hypothetical protein	Whole

				SPN23F_12620	Zeta toxin	Whole
				SPN23F_12630	Epsilon antitoxin	Whole
9	1257543	1270330	12788	SPN23F_12840	Conjugal transfer protein	Part
				SPN23F_12850	Conjugal transfer protein	Whole
				SPN23F_12860	Hypothetical protein	Whole
				SPN23F_12870	Hypothetical protein	Whole
				SPN23F_12880	Hypothetical protein	Whole
				traG	Conjugal transfer protein TraG	Whole
				SPN23F_12900	Hypothetical protein	Whole
				SPN23F_12910	Hypothetical protein	Whole
				SPN23F_12920	Hypothetical protein	Whole
				SPN23F_12930	Hypothetical protein	Whole
				SPN23F_12940	Conjugative transposon protein	Whole
				SPN23F_12950	Conjugative transposon protein	Whole
				SPN23F_12960	Conjugative transposon FtsK/SpoIIIE-family protein	Whole
				SPN23F_12970	Conjugative transposon replication initiation factor	Part
10	1280998	1288962	7965	SPN23F_13060	Conjugative transposon regulatory protein	Part
				SPN23F_13061	Hypothetical protein	Whole
				SPN23F_13070	Conjugative transposon regulatory protein	Whole
				xis	Excisionase	Whole
				SPN23F_13110	CAAX amino terminal protease	Whole
				SPN23F_13120	Hypothetical protein	Whole
				SPN23F_13130	Hypothetical protein	Whole
				SPN23F_13140	Hypothetical protein	Whole
				SPN23F_13150	DNA methylase	Whole
				SPN23F_13160	Replication initiator protein	Whole
				SPN23F_13170	Hypothetical protein	Whole
				rplL	50S ribosomal protein L7/L12	Part
11	1526952	1559263	32312	SPN23F_15810	Hypothetical protein	Whole
				SPN23F_15820	Hypothetical protein	Whole
				SPN23F_15830	Hypothetical protein	Whole
				SPN23F_15840	Endoribonuclease L-PSP family protein	Whole
				engB	Ribosome biogenesis GTP-binding protein YsxC	Whole
				clpX	ATP-dependent protease ATP-binding subunit ClpX	Whole
				SPN23F_15870	Hypothetical protein	Whole
				dyr	Dihydrofolate reductase	Whole
				dpr	Dps-like peroxide resistance protein Dpr	Whole
				SPN23F_15900	Exported choline-binding glycosyl hydrolase	Whole
				tpiA	Triosephosphate isomerase	Whole

						(1 nucleotide substitution at position 1536099)
				SPN23F_15920	DNA replication protein DnaD	Whole
				metA	Homoserine O-succinyltransferase	Whole
				apt	Adenine phosphoribosyltransferase	Whole
				SPN23F_15950	Methyltransferase	Whole
				SPN23F_15960	Hypothetical protein	Whole
				msmK	Multiple sugar-binding transport ATP-binding protein	Whole
				SPN23F_15990	Isochorismatase family protein	Whole
				codY	Transcriptional repressor CodY	Whole
				SPN23F_16010	DEAD/DEAH box helicase	Whole
				SPN23F_16020	Major facilitator superfamily protein	Whole
				SPN23F_16030	Pyridine nucleotide-disulfide oxidoreductase	Whole
				SPN23F_16040	Mur ligase family protein	Whole
				SPN23F_16050	Aminotransferase	Whole
				pepQ	Xaa-Pro dipeptidase	Whole
				SPN23F_16070	GNAT family acetyltransferase	Whole
				SPN23F_16080	IS3-Spn1 ORF B	Whole (1 nucleotide substitution at position 1551575)
				SPN23F_16100	Hypothetical protein	Whole
				thiD	Phosphomethylpyrimidine kinase	Whole
				truA	tRNA pseudouridine synthase A	Whole
				SPN23F_16140	Major facilitator superfamily protein	Whole
				SPN23F_16150	Hypothetical protein	Whole
				SPN23F_16160	PhnA protein	Whole
				cmk	Cytidylate kinase	Whole
				SPN23F_16180	Hypothetical protein	Whole
				SPN23F_16190	Hypothetical protein	Whole
				SPN23F_16200	Membrane glycosyl transferase	Part
12[a]	1584029	1589382	5354	SPN23F_16480	Metallo-beta-lactamase superfamily protein	Whole
				pepO	Endopeptidase O	Whole
				mtsB	Metal cation ABC transporter ATP-binding protein	Whole
				mtsC	Metal cation ABC transporter membrane protein	Whole
				mtsA	Metal ABC transporter substrate-binding lipoprotein precursor	Part
13	1612934	1618295	5362	*pbp2b*	Penicillin-binding protein 2b	Part
				SPN23F_16750	Transcription regulator	Whole
				SPN23F_16760	ROK family protein	Whole
				nanH	N-acetylneuraminate lyase	Whole

				SPN23F_16780	Hypothetical protein	Whole
14[a]	1994357	2005122	10766	*tkt*	Transketolase	Part
				SPN23F_20520	L-ascorbate 6-phosphate lactonase	Whole
				SPN23F_20530	Transcriptional antiterminator	Whole
				araD	L-ribulose-5-phosphate 4-epimerase	Whole
				ulaD	3-keto-L-gulonate-6-phosphate decarboxylase	Whole
				SPN23F_20580	Sugar phosphotransferase system (PTS), IIA component	Whole
				SPN23F_20590	Sugar phosphotransferase system (PTS), lactose/cellobiose-specific family, IIB subunit protein	Whole
				SPN23F_20600	PTS system ascorbate-specific transporter subunit IIC	Whole
				SPN23F_20610	Hypothetical protein	Whole
				SPN23F_20620	ssDNA-binding protein	Whole
				SPN23F_20630	Membrane protein OxaA 1 precursor	Whole
				rnpA	Ribonuclease P	Part
15	2005748	2024932	19185	*ackA*	Acetate kinase	Part
				SPN23F_20670	Hypothetical protein	Whole
				SPN23F_20700	Hypothetical protein	Whole
				SPN23F_20710	Hypothetical protein	Whole
				SPN23F_20720	Hypothetical protein	Whole
				SPN23F_20730	Competence protein	Whole
				comYC	Competence protein	Whole
				SPN23F_20750	Competence protein	Whole
				SPN23F_20760	Competence protein	Whole
				SPN23F_20770	Hypothetical protein	Whole
				SPN23F_20780	Zinc-binding alcohol dehydrogenase	Whole
				SPN23F_20790	N-acetylglucosamine-6-phosphate deacetylase	Whole
				SPN23F_20800	Acyltransferase family protein	Whole
						1 nucleotide substitution at position 2017038
				tgt	Queuine tRNA-ribosyltransferase	Whole
				SPN23F_20820	Hypothetical protein	Whole
				SPN23F_20830	Pyrrolidone-carboxylate peptidase	Whole
				SPN23F_20840	Hypothetical protein	Whole
				SPN23F_20850	MarR family transcriptional regulator	Whole
				SPN23F_20870	Hypothetical protein	Whole
				SPN23F_20880	Haloacid dehalogenase-like hydrolase	Whole
				SPN23F_20890	Multi antimicrobial extrusion (MATE) family transporter	Whole
				SPN23F_20900	Threonine synthase	Part

CDS = coding sequences. [a]Region also identical or highly similar in one or more CC66 representative.

Table S5. Regions of the PMEN3 genome identified as putatively recombinogenic and acquired from PMEN1.

Region	PMEN1-reference coordinates		Size (bp)	Included CDS	Predicted function	CDS in whole or part
	Start	End				
1	272645	294748	22104	SPN23F_02870	Glycosylhydrolase	Part
				SPN23F_02880	Glutathione peroxidase	Whole
				SPN23F_02890	Hyaluronate lyase	Whole
				kgdA	Keto-hydroxyglutarate-aldolase/keto-deoxy- phosphogluconate aldolase	Whole
				SPN23F_02920	PfkB family carbohydrate kinase	Whole
				SPN23F_02930	Hypothetical protein	Whole
				idnO	Gluconate 5-dehydrogenase	Whole
						1 nucleotide substitutions at positions 282302<br>1 nucleotide insertion in PMEN3 at position 282417
				SPN23F_02950	N-acetylgalactosamine-specific phosphotransferase system (PTS), IIA component	Whole
				ugl	Unsaturated glucuronyl hydrolase	Whole
				agaV	N-acetylgalactosamine-specific phosphotransferase system (PTS), IIB component	Whole
				agaW	N-acetylgalactosamine-specific phosphotransferase system (PTS), IIC component	Whole
				agaD	N-acetylgalactosamine-specific phosphotransferase system (PTS), IID component	Whole
				SPN23F_03000	Hypothetical protein	Whole
				SPN23F_03010	Heparinase II/III-like protein	Whole
				SPN23F_03020	LacI family transcriptional regulator	Whole
				SPN23F_03030	Membrane protein	Whole
				SPN23F_03040	Hypothetical protein	Whole
				SPN23F_03050	DNA-binding protein	Whole
				mraW	S-adenosyl-methyltransferase MraW	Whole
				ftsL	Cell division protein	Whole
				pbp2x	Penicillin-binding protein 2x	Whole
				mraY	Phospho-N-acetylmuramoyl-pentapeptide- transferase	Part
2	324107	346147	22041	*aliA*	Extracellular oligopeptide-binding protein	Part
				SPN23F_03400	Cell wall surface anchored protein	Whole
				pbp1A	Penicillin-binding protein 1A	Whole
				recU	Holliday junction-specific endonuclease	Whole

				SPN23F_03430	Hypothetical protein	Whole
				SPN23F_03440	DivIVA protein	Whole
				SPN23F_03450	RNA methylase family protein	Whole
				SPN23F_03460	Hypothetical protein	Whole
				gnd	6-phosphogluconate dehydrogenase	Whole
				csrR	Response regulator protein	Whole
				cbpJ	Choline binding protein J	Whole
				SPN23F_03500	Major facilitator superfamily protein	Whole
				mvaK1	Mevalonate kinase	Whole
				mvaD	Mevalonate diphosphate decarboxylase	Part
3	507199	520268	13070	*pheT*	Phenylalanyl-tRNA synthetase subunit beta	Part
				SPN23F_05260	Integral membrane protein (possible nuclease activity)	Whole
				SPN23F_05280	Hypothetical protein	Whole
				metE	5-methyltetrahydropteroyltriglutamate-- homocysteine S-methyltransferase	Whole
				metF	5,10-methylenetetrahydrofolate reductase	Whole
				pnpA	Polynucleotide phosphorylase/polyadenylase	Whole
				cysE	Serine acetyltransferase	Whole
				SPN23F_05330	GNAT family acetyltransferase	Whole
				cysS	Cysteinyl-tRNA synthetase	Whole
				SPN23F_05350	Ribonuclease	Whole
				SPN23F_05360	Leucine-rich protein	Part
4	634685	642400	7716	*thiE*	Thiamine-phosphate pyrophosphorylase	Part
				SPN23F_06440	Hypothetical protein	Whole
				SPN23F_06450	ABC transporter ATP-binding protein	Whole
				SPN23F_06460	Hypothetical protein	Whole
				SPN23F_06470	Coenzyme	Whole
				SPN23F_06480	Hypothetical protein	Whole
				SPN23F_06490	Hydroxyethylthiazole kinase	Whole
				thiE	Thiamine-phosphate pyrophosphorylase	Whole
				thiD	Phosphomethylpyrimidine kinase	Whole
				SPN23F_06520	Regulator	Part
5[a]	1598816	1615831	17016	*ileS*	Isoleucyl-tRNA synthetase	Part
				SPN23F_16620	Cell-division protein DivIVA	Whole
				SPN23F_16630	Hypothetical protein	Whole
				SPN23F_16640	Hypothetical protein	Whole
				SPN23F_16650	Hypothetical protein	Whole
				SPN23F_16660	Hypothetical protein	Whole
				ftsZ	Cell division protein FtsZ	Whole

				ftsA	Cell division protein	Whole
				SPN23F_16690	Hypothetical protein	Whole
				SPN23F_16700	MutT/NUDIX hydrolase family protein	Whole
				murF	UDP-N-acetylmuramoyl-tripeptide--D-alanyl-D- alanine ligase	Whole
				ddl	D-alanyl-alanine synthetase A	Whole
				recR	Recombination protein RecR	Whole
				pbp2b	Penicillin-binding protein 2b	Whole
				SPN23F_16750	Transcription regulator	Whole
				SPN23F_16760	ROK family protein	Part
6[b]	2033600	2038261	4662	*gltX*	Glutamyl-tRNA synthetase	Whole
				pgi	Glucose-6-phosphate isomerase	Whole
				SPN23F_20970	Peptidase	Whole
				SPN23F_20980	ABC transporter ATP-binding protein	Part
7[b]	2139058	2147857	8800	SPN23F_21910	Exported glycosyl hydrolase	Part
				SPN23F_21920	Hypothetical protein	Whole
				SPN23F_21930	PTS system, IId component	Whole
				SPN23F_21940	PTS system, IIc component	Whole
				SPN23F_21950	PTS system, mannose-specific IIAB component	Whole
				SPN23F_21960	PTS system, IIa component	Whole
				fucU	Fuscose operon FucU protein	Whole[c]
				fucA	L-fuculose phosphate aldolase	Whole
				fucK	Fuculokinase	Whole
				SPN23F_22000	Fucose phosphotransferase system repressor	Whole
8	2159726	2171248	11523	SPN23F_22150	Hypothetical protein	Whole
				SPN23F_22160	Hypothetical protein	Whole
				glpF1	Glycerol uptake facilitator protein 1	Whole
				glpO	Alpha-glycerophosphate oxidase	Whole
				glpK	Glycerol kinase	Whole
				SPN23F_22210	DNA-binding protein	Whole
				hslO	Hsp33-like chaperonin	Whole
				SPN23F_22230	tRNA-dihydrouridine synthase	Whole
				cbpA	Choline-binding surface protein A	Part
9	2171249	2182005	10757	*cbpA*	Choline-binding surface protein A	Part
				SPN23F_22250	Isoprenylcysteine carboxyl methyltransferase (ICMT) family protein	Whole
				SPN23F_22260	Two-component system, sensor histidine kinase	Whole
				SPN23F_22270	Two-component system, response regulator	Whole
				clpC	Stress response-related Clp ATPase	Whole
				ctsR	Transcriptional regulator	Whole
				SPN23F_22300	ABC transporter ATP-binding protein	Whole

	SPN23F_22310	ABC transporter substrate-binding protein	Whole
	SPN23F_22320	ABC transporter permease	Whole
	SPN23F_22330	Hypothetical protein	Whole
	cbpD	Choline binding protein D	Part

CDS = coding sequences. [a]One or more CC66 representatives are also identical over the first ~14 kb of this region. [b]Region also identical or highly similar in one or more CC66 representative. [c]A duplication of 359 bp of the *fucU* gene was identified directly 5' of the full version of this gene in the PMEN3 genome. The sequence was identical to the equivalent region of the PMEN1 reference genome and differed from that of the PMEN3 ancestral representatives, suggesting that this duplication occurred subsequent to the import of this region from PMEN1.
