# Supplementary material for: The multidrug-resistant PMEN1 pneumococcus is a paradigm for genetic success
Source: Genome Biol. 2012 Nov 16;13(11):R103. doi: 10.1186/gb-2012-13-11-r103 (PMC3580495; doi:10.1186/gb-2012-13-11-r103)
Supplement: Additional file 3 — Table S6. More information (for example, serotype and penicillin susceptibility) about the isolates for which whole-genome sequences were analyzed in this study. [file gb-2012-13-11-r103-S3.PDF]

**Table S6. Isolates for which whole-genome sequences were analysed in this study.**

| Isolate | Other name(s) | Serotype | Year | Country | CC | Penicillin susceptibility[c] | Genbank accession no. |
|---|---|---|---|---|---|---|---|
| 14/5[a] | | 14 | 1967 | Denmark | 15 | S | |
| PMEN10[a] | CSR[14]-10; ATCC700677 | 14 | 1987 | CSR | 15 | R | |
| PMEN9[a] | England[14]-9; ATCC700676 | 14 | 1993 | England | 15 | S | |
| SPnINV200[b] | | 14 | 1995 | England | 15 | S | FQ312029.1 |
| ICE13[a] | | 14 | 1998 | Iceland | 15 | S | |
| Ala243[a] | L2003-01243 | 14 | 1998 | USA | 15 | I | |
| Ala317[a] | L2003-01317 | 14 | 2001 | USA | 15 | I | |
| ICE50[a] | | 14 | 2003 | Iceland | 15 | I | |
| CGSP14[b] | | 14 | 2004-2005 | Taiwan | 15 | R | NC_010582.1 |
| PMEN5[a] | Spain[14]-5; ATCC700902 | 14 | 1990 | Spain | 18 | I | |
| MLV-016[b] | | 11A | Unknown | Unknown | 62 | Unknown | ABGH01000001-ABGH01000126 |
| 7B/2[a] | | 7B | 1952 | USA | 66 | S | |
| 9N/6[a] | | 9N | 1960 | Denmark | 66 | S | |
| 19F/11[a] | | 19F | 1972 | Denmark | 66 | S | |
| JJA[b] | | 14 | 1995 | Brazil | 66 | I | CP000919.1 |
| PMEN18[a] | Tennessee[14]-18; ATCCBAA-340 | 14 | 1997 | USA | 66 | R | |
| USA9[a] | PATH#183 | 14 | 1999 | USA | 66 | I | |
| USA8[a] | 0680-02 | 9N | 2001 | USA | 66 | S | |
| USA12[a] | PATH#678 | 23F | 2001 | USA | 66 | S | |
| USA13[a] | PATH#688 | 19F | 2005 | USA | 66 | S | |
| USA11[a] | PATH#286 | 14 | Unknown | USA | 66 | S | |
| PMEN1[b] | Spain[23F]-1; ATCC700669 | 23F | 1984 | Spain | 81 | I | NC_011900.1 |
| PMEN12[a] | Finland[6B]-12; ATCC700903 | 6B | 1987 | Finland | 90 | R | |
| PMEN2[a] | Spain[6B]-2; ATCC 700670 | 6B | 1988 | Spain | 90 | I | |
| 670[b] | | 6B | 1988 | Spain | 90 | Unknown | CP002176.1 |
| PMEN22[a] | Greece[6B]-22; ATCCBAA-658 | 6B | 1995 | Greece | 90 | S | |
| 17F/2[a] | | 17F | 1939 | Denmark | 113 | S | |
| 18C/2[a] | | 18C | 1939 | Denmark | 113 | S | |

| | | | | | | | |
|---|---|---|---|---|---|---|---|
| 18B/2[a] | 1033/41 (da) | 18B | 1941 | Denmark | 113 | S | |
| 35C/2[a] | | 35C | 1941 | Denmark | 113 | S | |
| 35C/3[a] | 7765/43 (da) | 35C | 1943 | Denmark | 113 | S | |
| 9V/4[a] | 980/68 | 9V | 1968 | Denmark | 113 | S | |
| 18C/3[a] | | 18C | 1968 | Denmark | 113 | S | |
| PMEN36[a] | Netherlands[18C]-36 | 18C | 1980 | Netherlands | 113 | S | |
| USA2[a] | 6223-99 | 18C | 1999 | USA | 113 | S | |
| ICE501[a] | | 18C | 2002 | Iceland | 113 | S | |
| 14/2[a] | 34359 | 14 | 1952 | USA | 124 | S | |
| 9L/2[a] | | 9L | 1952 | USA | 124 | S | |
| 11C/1[a] | Eddy nr. 53 | 11C | 1957 | USA | 124 | S | |
| 14/4[a] | | 14 | 1961 | Denmark | 124 | S | |
| PMEN35[a] | Netherlands[14]-35 | 14 | 1980 | Netherlands | 124 | S | |
| 14/9[a] | | 14 | 1982 | Denmark | 124 | S | |
| 14/7[a] | | 14 | 1992 | Denmark | 124 | S | |
| Ala292[a] | L2003-01292 | 14 | 1998 | USA | 124 | S | |
| USA6[a] | 4347-99 | 14 | 1999 | USA | 124 | S | |
| Ala289[a] | L2003-01289 | 14 | 2002 | USA | 124 | S | |
| Ala263[a] | L2003-01263 | 14 | 2002 | USA | 124 | S | |
| ICE46[a] | | 14 | 2003 | Iceland | 124 | S | |
| ICE594[a] | | 14 | 2005 | Iceland | 124 | S | |
| SPnOXC141[b] | | 3 | 2001 | England | 180 | S | FQ312027.1 |
| 7A/2[a] | 2040/37 | 7A | 1937 | Denmark | 191 | S | |
| 7F/3[a] | | 7F | 1962 | Denmark | 191 | S | |
| PMEN39[a] | Netherlands[7F]-39 | 7F | 1984 | Netherlands | 191 | S | |
| 7F/4[a] | | 7F | 1986 | Scotland | 191 | S | |
| ICE22[a] | | 7F | 1993 | Iceland | 191 | S | |
| CDC1087-00[b] | | 7F | 1999 | USA | 191 | S | ABFT01000001-ABFT01000075 |
| USA16[a] | PATH#224 | 7F | 2003 | USA | 191 | S | |
| CDC3059-06[b] | | 19A | 2005 | USA | 199 | I | ABGG01000001-ABGG01000033 |
| TIGR4[b] | | 4 | 1991 | Norway | 205 | Unknown | AE005672.3 |
| P1031[b] | | 1 | 2002 | China | 217 | S | CP000920.1 |
| 7F/2[a] | | 7F | 1952 | USA | 218 | S | |
| 12F/5[a] | | 12F | 1988 | Denmark | 218 | S | |

| | | | | | | | |
|---|---|---|---|---|---|---|---|
| ICE23[a] | | 7F | 1993 | Iceland | 218 | S | |
| PMEN34[a] | Denmark$^{12F}$-34 | 12F | 1995 | Canada | 218 | S | |
| 12F/6[a] | | 12F | 1996 | Denmark | 218 | S | |
| USA18[a] | 4621-00 | 12F | 1999 | USA | 218 | S | |
| USA20[a] | PATH#129 | 7F | 1999 | USA | 218 | S | |
| CDC0288-04[b] | | 12F | 2003-2004 | Unknown | 218 | Unknown | ABGF01000001-ABGF01000038 |
| PMEN15[a] | Taiwan$^{23F}$-15; ATCC700906 | 23F | 1997 | Taiwan | 242 | I | |
| 70585[b] | | 5 | Unknown | Unknown | 289 | Unknown | CP000918.1 |
| SPnINV104[b] | | 1 | 1998 | England | 306 | S | FQ312030.1 |
| PMEN17[a] | Maryland$^{6B}$-17; ATCCBAA-342 | 6B | 1997 | USA | 385 | I | |
| 17F/3[a] | | 17F | 1962 | Denmark | 392 | S | |
| 17F/4[a] | | 17F | 1962 | Denmark | 392 | S | |
| 23A/2[a] | 1196/45 | 23A | 1945 | Denmark | 439 | S | |
| 23F/5[a] | | 23F | 1979 | Germany | 439 | S | |
| PMEN4[a] | Tennessee$^{23F}$-4; ATCC51916 | 23F | 1991 | USA | 439 | I | |
| 23F/10[a] | | 23F | 1996 | Denmark | 439 | S | |
| 22A/2[a] | 3405/39(da) | 22A | 1939 | Denmark | 490 | S | |
| 2/3[a] | | 2 | 1943 | Denmark | 490 | S | |
| 10F/2[a] | 34355 | 10F | 1956 | USA | 490 | S | |
| 18F/1[a] | | 18F | 1961 | USA | 490 | S | |
| ICE11[a] | | 6B | 1998 | Iceland | 490 | S | |
| USA22[a] | 3140-06 | 6C | 2005 | USA | 490 | I | |
| 14/8[a] | | 14 | 1976 | Denmark | 554 | I | |
| 17F/1[a] | | 17F | 1952 | USA | 574 | S | |
| 2/2[a] | pn2L | 2 | 1956 | USA | 574 | S | |
| 14/3[a] | | 14 | 1939 | Denmark | 1106 | S | |
| CDC1873-00[b] | | 6A | 1999 | USA | 2090 | R | ABFS01000001-ABFS01000053 |
| 19F/8[a] | | 19F | 1952 | Denmark | 4399 | S | |
| 12F/3[a] | | 12F | 1961 | Denmark | 4399 | S | |
| 19F/5[a] | | 19F | 1962 | Denmark | 4399 | S | |
| 9A/1[a] | | 9A | 1962 | USA | 156/162 | S | |
| 9V/5[a] | | 9V | 1991 | Denmark | 156/162 | S | |
| PMEN3[a b] | Spain$^{9V}$-3; ATCC 700671; | 9V | 1993 | France | 156/162 | I | ABGE01000001- |

| | | | | | | | |
|---|---|---|---|---|---|---|---|
| | SP195 | | | | | | ABGE01000041 |
| 9V/6[a] | | 9V | 1994 | Denmark | 156/162 | S | |
| PMEN14[a] | Taiwan[19F]-14; ATCC700905 | 19F | 1997 | Taiwan | 271/320 | R | CP000921.1 |
| ICE27[a] | | 19F | 1995 | Iceland | 422/476 | S | |
| D39[b] | | 2 | 1916 | Unknown | None128 | Unknown | CP000410.1 |
| R6[b] | | NT | 1964 | Unknown | None128 | Unknown | AE007317.1 |
| 9N/2[a] | | 9N | 1952 | USA | None3782 | S | |
| PMEN13[a] | S.Africa[19A]-13; ATCC700904 | 19A | 1988 | South Africa | None41 | I | |
| 23F/4[a] | | 23F | 1967 | Australia | Singleton10030 | I | |
| 12F/2[a] | | 12F | 1962 | USA | Singleton10043 | S | |
| 19F/10[a] | | 19F | 1963 | Denmark | Singleton10087 | S | |

[a]Isolate subjected to whole-genome sequencing in this study. [b]Genome retrieved from Genbank. [c]S = Susceptible, MIC ≤0.06 μg/ml, I = Intermediate, MIC 0.12 - 1 μg/ml, R = Resistant, MIC ≥2.0 μg/ml.
