# Supplementary material for: The multidrug-resistant PMEN1 pneumococcus is a paradigm for genetic success
Source: Genome Biol. 2012 Nov 16;13(11):R103. doi: 10.1186/gb-2012-13-11-r103 (PMC3580495; doi:10.1186/gb-2012-13-11-r103)
Supplement: Additional file 4 — Supporting Results and Methods. More information about MLST and pbp diversity, the imported regions of CGSP14 and PMEN3, coding sequences found in both PMEN1 and isolates of CC66, outlier isolates highlighted in Figure 4 and methodological details related to whole-genome sequence assemblies. [file gb-2012-13-11-r103-S4.PDF]

Supporting Results and Methods for "The Multidrug-resistant PMEN1 Pneumococcus: A Paradigm for Genetic Success"

MLST and pbp diversity

232 unique STs and 163 CCs were represented by isolates in this study. (Note that after repeated attempts using multiple primer sets, the *ddl* sequence for isolate SA82 (1989, CC124[14]) could not be determined, presumably due to sequence divergence within the primer-binding region.) Among the 389 isolates selected for *pbp* sequencing plus the 16 genomes, 401, 389 and 404 *pbp2x, pbp1a* and *pbp2b* sequences, respectively, were obtained (a full complement of sequences could not be obtained for all isolates due to PCR and/or sequencing difficulties most likely also arising from sequence divergence in the primer binding regions). Among these sequences 141, 104 and 127 unique *pbp2x, pbp1a* and *pbp2b* alleles were identified, respectively, and of these 64 (45.4%), 33 (31.7%) and 59 (46.5%) were altered (i.e. >1% divergent from the penicillin-susceptible R6 reference strain dated 1964). There was no evidence of PNSP or altered *pbps* prior to 1967. 18/64 *pbp2x*, 5/33 *pbp1a* and 17/59 *pbp2b* altered alleles contained regions which spanned ≥1 active site coding region and were identical or highly similar (≤2 nucleotide substitutions) to those of the PMEN1 reference strain [see Additional file 1]. There were other regions of *pbp* sequence that were shared among different alleles, which was expected given the well-recognised mosaic nature of pneumococcal *pbps*; however, we could find no example of *pbp* alleles (or parts of *pbp* alleles) that were more frequent than those of PMEN1, in fact, none were even close to being as frequent.

pbp sequences from 233 of 237 (98.3%) PSP were ≤1% divergent from those of R6. *pbp2x* sequences from four PSP were between 3.7% and 9.6% divergent from those of R6. Three of

these isolates were PMEN reference strains (PMEN21, PMEN30 and PMEN40) and the fourth was a CC66[14] representative (USA11). To our knowledge, possession of altered *pbp* sequences without increased penicillin MIC has not been noted previously for pneumococci. One possible explanation for this is that the penicillin MIC was not increased for these pneumococci due to the lack of altered *pbp1a* and/or *pbp2b* sequences and/or favourable alleles at other loci believed to play a role in penicillin resistance. Alternatively, *pbp2x* sequence divergence may have followed acquisition and recombination of DNA from penicillin-susceptible viridans streptococci, resulting in sequence mosaics lacking the appropriate nucleotide changes to confer resistance.

***pbp* diversity within the PMEN1 lineage**

Within the PMEN1 lineage there was some variation of *pbp* alleles: 1, 2 and 2 of the 28 isolates in our collection possessed alternative *pbp2x, pbp1a* and/or *pbp2b* alleles respectively (i.e. these alleles were not identical to those of the PMEN1 reference strain). However, all but one of these alleles (the *pbp2x* allele) did contain region(s) which were identical or highly similar to those of the reference strain alleles.

CGSP14 and PMEN3 reference imported regions: search for identical regions among other pneumococci.

In order to assess whether the putatively imported regions of the CGSP14 and PMEN3 reference genomes could have in fact been acquired from a non-PMEN1 representative pneumococcus, we searched for these regions among our collection of pneumococcal genomes. While it is not possible to represent all of the potential pneumococcal genetic diversity, our collection does represent a diverse range of CCs including many of the major

global clones. Genome Comparator was used to identify coding sequences identical to those of the PMEN1 reference genome, the subsequent output was ordered by position in the PMEN1 reference chromosome and viewed in a spread sheet format. None of the putative imported regions were identical or highly similar across their whole length among other pneumococci in our collection, excluding isolate 23F/4 and CC66 representatives. In some cases similarities over parts of the regions could be identified, and we cannot exclude the possibility that these 'sub-regions' could have been acquired by CGSP14 or PMEN3 from a non-PMEN1 representative pneumococcus. However, if this were true the remaining 'sub-region(s)' would need to have been acquired independently via a secondary recombination event(s). In such an instance the most parsimonious solution, which we favour, is that the region was acquired as a single whole from a PMEN1 representative.

Isolate 23F/4 is an ancestral representative of PMEN1 and thus it is not surprising that some of the CGSP14 and PMEN3 putative imported regions may be highly similar in 23F/4 [see Figure below]. CC66 was considered to be closely related to PMEN1 (see below) and thus it is also not surprising that regions of the genomes of CC66 members were highly similar to the CGSP14 and PMEN3 putative imported regions. However, when the nucleotide sequences of the regions in question were viewed, the PMEN1 reference was clearly the best match in all but two and three cases respectively, supporting the hypothesis that the majority of the described regions were most likely acquired from a PMEN1 representative. Regarding the remaining five regions, we cannot be certain whether they were acquired from PMEN1 or CC66, but since these are the minority we do not believe that this result greatly affects our conclusions.

Finally, to assess whether our analyses could be biased by use of the PMEN1 as the Genome Comparator reference, we compared PMEN1, CGSP14, PMEN3 and all available members of their respective CCs using a CC66 representative (JJA) as the reference. We identified 104 and 40 coding sequences within the CGSP14 and PMEN3 genomes, respectively, which were identical to those of JJA but differed from those of other members of their respective CCs. 12 and 7 of these coding sequences, respectively, also differed from those of PMEN1. Using the same output we identified 197 and 109 coding sequences within the CGSP14 and PMEN3 genomes respectively which were identical to those of PMEN1 but differed from those of other members of their respective CCs. 105 and 76 of these respectively also differed from those of JJA. Therefore, the extent to which CGSP14 and PMEN3 may have acquired DNA from CC66 representatives is likely much less than that acquired from PMEN1 representatives and supports our previous findings.

A high proportion of coding sequences identified from CC66 representatives were identical to those of the PMEN1 reference strain.

Nine isolates from our collection represented CC66 and were selected for whole-genome sequencing. The genome of an additional CC66 isolate, JJA (unknown year of isolation but presumed from 1990 onwards, ST66[14]), was retrieved from Genbank (accession CP000919.1). Together, these isolates represented five independent serotypes (7B (n = 1), 23F (n = 1), 9N (n = 2), 19F (n = 2) and 14 (n = 4)), and five different STs (7180 (n = 1), 8119 (n = 1), 67 (n = 1), 71 (n = 2) and 66 (n = 6)). Among the CC66 isolate coding sequences identified by Genome Comparator, between 44.5% and 58.4% were shared identically with the PMEN1 reference. The three highest percentages (57.3%, 58.4% and 55.0%, respectively) were associated with the three oldest isolates (7B/2 (1952, ST7180[7B]), 9N/6

(1960, ST71^{9N}) and 19F/11 (1972, ST71^{19F}), respectively. Two of these isolates pre-dated the first identified PMEN1 representative (from 1984) and the predicted emergence of the PMEN1 clone (~1970), thus this observation is consistent with our suggestion that the similarities between these clones are due to ancestral descent rather than recombinogenic transfer of genetic material from PMEN1. However, PMEN18, a member of CC66, possessed *pbp2b* sequence regions highly similar to those of PMEN1 and these *pbp2b* regions were more likely to have been acquired through recent recombination events, owing to their absence among older CC66 isolates.

An alternative explanation for the increased number of identical coding sequences between PMEN1 and CC66 compared to other CCs could be that the ancestor of these CC66 isolates acquired identical copies of the large PMEN1 reference-associated mobile genetic elements from an independent source, artificially inflating the percentages of identical coding sequences reported here; however, we did not find that to be case. The largest of these elements was ICE*Sp*ST8123F, which consisted of 73 coding sequences, only 5 and 11 of which were identified identically from isolates USA11 (Unknown year of isolation but presumed from 1990 onwards, ST66^{14}) and PMEN18 (1997, ST67^{14}) respectively. The other large PMEN1 reference mobile elements included the ϕMM1 phage (50 coding sequences) and the *psrP* element (20 coding sequences). The ϕMM1 phage also was largely absent from the CC66 isolates, with only 1, 3 and 6 identical coding sequences identified from USA11, 7B/2 and USA12 (2001, ST66^{23F}) respectively. Additionally, only one to four PMEN1 reference *psrP* element identical coding sequences were identified from 9 of the 10 CC66 isolates, with none identified from the tenth isolate.

Brief comments on outlier isolates that were highlighted in Figure 4 (apart from strain 23F/4, which is discussed in the manuscript text).

Isolate CDC3059-06 (CC199, Genbank Accession ABGG01000001-ABGG01000033) shared 33.8% identical coding sequences with PMEN1, but without additional CC199 isolates or ancestral representatives we were unable to make any further inferences in this study. CGSP14 and PMEN3 shared 25.1% and 20.6% identical coding sequences with PMEN1, respectively, which was higher than their respective ancestral representatives and consistent with the findings detailed in the manuscript text. CDC0288-04 and USA18 both shared 20.0% identical coding sequences with PMEN1, potentially also due to recombination with PMEN1, although we cannot be certain as identical regions also matched those of CC66 representatives.

Additional methodological details related to the whole-genome sequencing data assemblies.

The Velvet optimiser script was used to optimise k-mer lengths to the longest length achieving an average (arithmetic mean) of ≥20x coverage across the assembled sequences when sufficient sequence data were available. Resulting average coverage values were isolate-dependent and ranged from 12x to 352x (note that all except one isolate, ICE570, had an average read coverage of ≥23x). As expected, read coverage was not constant across the entire length of each genome, as some sequence regions were more difficult to sequence/assemble and thus had a lower than average read coverage. Conversely, some regions such as those containing repeats of greater length than the sequence reads or those which were present in more than one copy within the genome, had a higher than average coverage. However, as part of its error removal processes, Velvet will remove assembly

contigs which fall below a minimum coverage cut-off value (minimum = 4), thus maintaining a constant level of accuracy across genomes and between isolates.

Whilst isolate-dependent coverage variation is not likely to significantly affect consensus sequence accuracy, it is known to affect assembly contig size [1]. Average coverage below ~20 - 30x was shown to result in reduced contig N50. Only a single isolate (ICE570) included in the analyses described in this work had an average coverage of <20x, resulting in an N50 value of 1,771 bp. The mean N50 for all isolates was ~43,000 bp. It is not likely that this variation would have significantly affected the findings discussed here since 1,771 bp is larger than the length of most pneumococcal genes and visual inspection of sequence alignments allowed for the detection and description of sequence gaps in regions of interest.

1. Zerbino, DR, Birney, E: **Velvet: algorithims for de novo short read assembly using de Bruijn graphs.** Genome Res 2008, **18**:821-829.

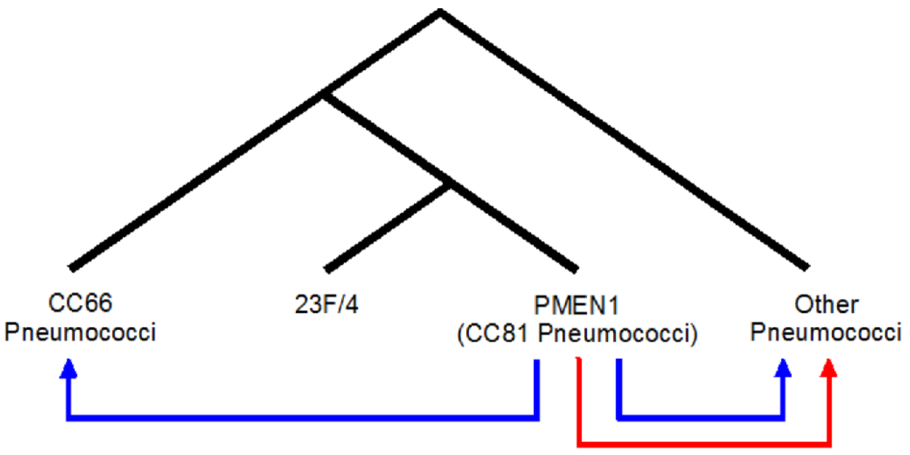

Figure S1: Idealised representation of the pneumococcal lineage relationships inferred from this study.

Blue arrows represent horizontal transfer of *pbp* nucleotide sequences between lineages. Red arrows represent horizontal transfer of other genomic regions between the PMEN1 lineage and alternate pneumococcal lineages (CC15 and CC156/162 described as examples in text).
