# Supplementary material for: The multidrug-resistant PMEN1 pneumococcus is a paradigm for genetic success
Source: Genome Biol. 2012 Nov 16;13(11):R103. doi: 10.1186/gb-2012-13-11-r103 (PMC3580495; doi:10.1186/gb-2012-13-11-r103)
Supplement: Additional file 5 — Figure S2. Depiction of the results of a Genome Comparator analysis of 104 pneumococcal genomes compared to the TIGR4 reference genome. [file gb-2012-13-11-r103-S5.PDF]

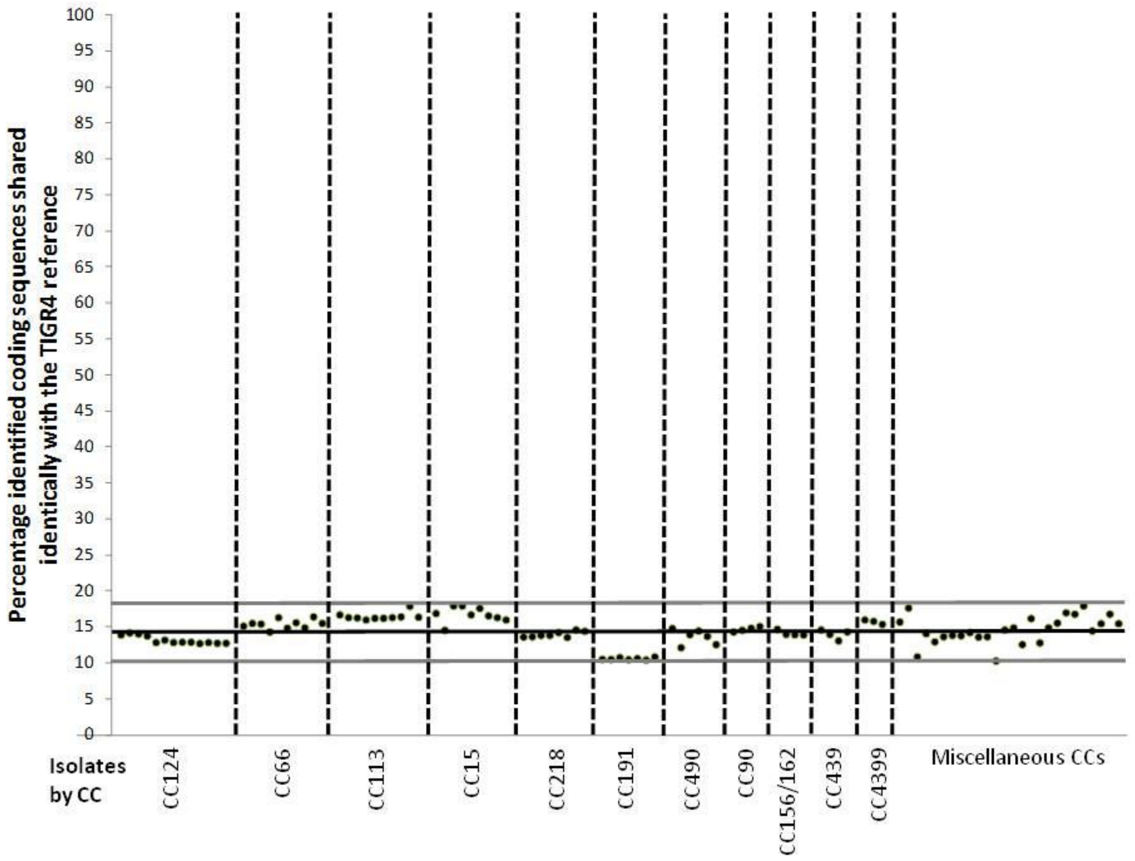

Figure S2: Percentage TIGR4 reference coding sequences identified identically within 104 pneumococcal genomes.

Points represent independent pneumococcal isolates. Isolates representing CCs for which n≥3 are labelled by CC. Isolates representing CCs for which n<3 are labelled as 'Miscellaneous CCs'. Black line represents mean percentage identical coding sequences ± 2 standard deviations (gray lines).
