# Supplementary material for: The multidrug-resistant PMEN1 pneumococcus is a paradigm for genetic success
Source: Genome Biol. 2012 Nov 16;13(11):R103. doi: 10.1186/gb-2012-13-11-r103 (PMC3580495; doi:10.1186/gb-2012-13-11-r103)
Supplement: Additional file 6 — Table S7. Summary of the 442 isolates included in this study, stratified by era of isolation. [file gb-2012-13-11-r103-S6.PDF]

Table S7. Summary of isolates included in this study stratified by era of isolation.

		Period of isolation										
		**1916**	**1937-39**	**1940s**	**1950s**	**1960s**	**1970s**	**1980s**	**1990s**	**2000s**	**Unknown**	**Total**
**Serotype**	1			4	1	1			5	1		12
	2	1		1	2							4
	3					5	2	1		1		9
	4				1	4			2			7
	5					1	1	2	1		1	6
	6						1					1
	6A				1		13	7	3	1		25
	6B		1					18	6			25
	6C									1		1
	7A		1			1						2
	7B				1				1			2
	7C		1				1					2
	7F				1	2		2	9	2		16
	8				1	3		1				5
	9A					1						1
	9L			1	1	1						3
	9N		1		1	3			1	1		7
	9V		1			1		6	3			11
	10A		1									1
	10B							1				1
	10C							1				1
	10F				1							1
	11A		1					1			1	3
	11B			1								1
	11C				1							1
	11D							1				1
	11F				1							1
	12A					12						12
	12B							1				1
	12F					3		1	4		1	9
	13				1							1
	14		1		1	3	5	7	19	13	1	50
	15A		1						1			2
	15B		1					1	1			3

15C				1						1
15F			1	1						2
16A						1				1
16F			1							1
17A					1					1
17F	1		1	2						4
18A			1							1
18B		1								1
18C	1	1		1		1	4	2		10
18F		1		1						2
19A	1		1	1	16	15	1	1		36
19B					1					1
19C	1									1
19F			3	5	1	4	5	1		19
20	1	1		1						3
21			1	1			1			3
22A	2									2
22F		1				1				2
23A		1								1
23B		1					1			2
23F		1		1	1	22	16	1		42
24A		1								1
24B		1								1
24F		1	1							2
25A						2				2
25F			1	2						3
27			1							1
28A		1								1
28F			1							1
29			1			3				4
31			2	1						3
32A		1								1
32F			1							1
33A	1	1								2
33B				1						1
33C		1								1
33D					2					2
33F	1									1
34			1	1	2					4

	35A		1									1
	35B		1			1	1		1			4
	35C			2								2
	35F		1									1
	36		1				1		2			4
	37			1		1	4		1			7
	38		1					1				2
	39			1								1
	40				1							1
	41A						2					2
	41F			1								1
	42						1					1
	43			1	1							2
	44				1							1
	45			2	2							4
	46				1							1
	47A						1					1
	47F						1					1
	48				3	2						5
	NT					1		2	1			4
**Penicillin Susceptibility**[a]	S		24	30	41	63	26	24	45	19	1	273
	I					2	7	57	24	4		94
	R						25	21	19	2		67
	Unknown	1			1	1		1	1		3	8
**Grand Total**		1	**24**	**30**	**42**	**66**	**58**	**103**	**89**	**25**	**4**	**442**[b]

[a]S = Susceptible, MIC ≤0.06 µg/ml, I = Intermediate, MIC 0.12 - 1 µg/ml, R = Resistant, MIC ≥2.0 µg/ml. [b]Including strains for which genomic sequences were retrieved from Genbank (n = 16).
