# Supplementary material for: The multidrug-resistant PMEN1 pneumococcus is a paradigm for genetic success
Source: Genome Biol. 2012 Nov 16;13(11):R103. doi: 10.1186/gb-2012-13-11-r103 (PMC3580495; doi:10.1186/gb-2012-13-11-r103)
Supplement: Additional file 7 — Table S8. Details of the primers used for PCR and conventional sequencing of regions of pbp2x, pbp1a and pbp2b. [file gb-2012-13-11-r103-S7.PDF]

Table S8. Primers for PCR and conventional sequencing of regions of *pbp2x, pbp1a* and *pbp2b.*

Gene	Primer name	Primer sequence (Forward primers (F) 5'-3', Reverse primers (R) 3'-5')	Application	Trimmed allele length (bp)
pbp2x	Sp36F	GAA GAA TCC TAT GTA AGA GAG C	PCR / sequencing	1673
	Sp35F	GAG GAC TTT GTT TGG CGT GA	Sequencing only	
	Sp34R	GCT GAA GCC CGC TCC AAG AT	Sequencing only	
	Sp33R	GCT GGC CTG TAA TTT GCG CCT	PCR / sequencing	
	Sp37F[a]	ACA GGT CAA ATA CTA CAG ATG C	PCR / sequencing	
	Sp52R[a]	CCT TGC ACA TAG GCA ATA ACT CC	PCR / sequencing	
	*pbp2x*F1[a]	GCT GAG GAC GCA ACY TCC	PCR / sequencing	
	*pbp2x*F2[a]	AAG GAC TTT GTT TGG CGT GA	PCR / sequencing	
	*pbp2x*F4[a]	ACT TCT GGG ATG GAG AGT TCC	PCR / sequencing	
	*pbp2x*R1[a]	GTG ATT CTT TCA TAG CTG AGG C	PCR / sequencing	
	*pbp2x*F5[a]	ACC GAA ATG GAG TCC CGA TTG C	PCR / sequencing	
	*pbp2x*R4[a]	ATC TCG AGT CGT CGC ATC CGC	PCR / sequencing	
pbp1a	Sp47F	ACT ATT ATT TGT GCT TGG AGT GGT TGA GC	PCR / sequencing	901
	Sp43R	CCA AGA AGC TCA AAA ACA TCT GTG GG	PCR / sequencing	
	*pbp1a*F[a]	TTG ATG GGG GTT GTT GTG GAG C	PCR / sequencing	
	pbp1aR2[a]	AGC CTT GCT GGC TGG AAT GC	PCR / sequencing	
	pbp1aR3[a]	GGC TAT AAC CTT CTA ACT ACT GGG	PCR / sequencing	
pbp2b	*pbp2b*_2F	GCG TAT AGT TAT CAA ACT GCC CA	PCR / sequencing	1272
	*pbp2b*_3F	ACC TGT TTT TGG GTT AAG GGC	Sequencing only	
	*pbp2b*_3R	CAA ATT TGA CAG AAC GCC AGC	Sequencing only	
	*pbp2b*_4R	ACA GCC ATT CGA TTC CGA TTC	PCR / sequencing	

[a]Alternative primers used following failure of initial primers and/or when sequences were suspected to be divergent in the primer binding regions.
