# Supplementary material for: The multidrug-resistant PMEN1 pneumococcus is a paradigm for genetic success
Source: Genome Biol. 2012 Nov 16;13(11):R103. doi: 10.1186/gb-2012-13-11-r103 (PMC3580495; doi:10.1186/gb-2012-13-11-r103)
Supplement: Additional file 8 — Table S9. Details of the metadata for the 442 isolates included in this study. [file gb-2012-13-11-r103-S8.PDF]

Table S9. Demographic, genotype and penicillin susceptibility information for the isolate collection used in this study.

Isolate	Other Name(s)	Serotype	Year	Country	Contributor	ST	CC	Penicillin MIC	Reference(s)	Genome Accession
1/2		1	1965	USA	LML	615	615	0.016		
1/3		1	1956	Denmark	LML	7241	615	0.023		
1/4	519/43	1	1943	Denmark	LML	5316	306	0.012	[2]	
1/5		1	1943	Denmark	LML	5316	306	0.012		
1/6		1	1944	Denmark	LML	5316	306	0.023		
1/7		1	1991	Denmark	LML	250	304	0.016		
2/2 *	pn2L	2	1956	USA	LML	574	574	0.016	[2]	ERR025820‡
2/3 *		2	1943	Denmark	LML	3744	490	0.032		ERR026715‡
2/4		2	1959	Denmark	LML	595	None128	0.023		
3/2		3	1973	USA	LML	378	378	0.012		
3/3		3	1971	USA	LML	7244	378	0.016		
3/4		3	1961	Denmark	LML	378	378	0.023		
3/5		3	1962	Denmark	LML	7169	2021	0.016		
3/6	524/62	3	1962	Denmark	LML	7211	1024	0.012	[2]	
3/7		3	1961	Denmark	LML	1377	378	0.023		
3/8		3	1961	Denmark	LML	1377	378	0.023		
4/2		4	1952	USA	LML	7224	Singleton7224	0.016		
4/3		4	1962	Denmark	LML	247	247	0.016		
4/4	600/62	4	1962	Denmark	LML	7223	247	0.016	[2]	
4/5		4	1962	Denmark	LML	249	247	0.023		
5/1	Ambrose	5	1976	USA	LML	4840	None3035	0.012	[2]	
5/2		5	1961	USA	LML	4840	None3035	0.016		
5/3		5	1987	Denmark	LML	289	289	0.023		
5/4		5	1987	Singapore	LML	7222	289	0.023		
6A/2	34351	6A	1952	USA	LML	7188	460	0.016	[2]	
6B/3	2616/39	6B	1939	Denmark	LML	7199	Singleton7199	0.008	[2]	
7A/2 *	2040/37	7A	1937	Denmark	LML	191	191	0.008	[2]	ERR025824‡
7A/3		7A	1968	USA	LML	5994	Singleton5994	0.032		
7B/2 *	Johnson	7B	1952	USA	LML	7180	66	0.023	[2]	ERR025825‡
7B/3		7B	1996	France	LML	230	230	0.500		
7C/2		7C	1939	Denmark	LML	7175	43	0.012		

Isolate	Other Name(s)	Serotype	Year	Country	Contributor	ST	CC	Penicillin MIC	Reference(s)	Genome Accession
7C/3	Sutcliff	7C	1971	USA	LML	7171	43	0.016	[2]	
7F/2 *		7F	1952	USA	LML	7210	218	0.023		ERR026172‡
7F/3 *		7F	1962	Denmark	LML	191	191	0.016		ERR026176‡
7F/4 *		7F	1986	Scotland	LML	7208	191	0.023		ERR026177‡
7F/5	554/62	7F	1962	Denmark	LML	191	191	0.012	[2]	
7F/6		7F	1996	Sweden	LML	7210	218	0.023		
8/2		8	1952	USA	LML	53	62	0.016		
8/3		8	1962	Denmark	LML	53	62	0.012		
8/4	573/62	8	1962	Denmark	LML	7203	1480	0.016	[2]	
8/5		8	1969	Belgium	LML	404	1480	0.016		
9A/1 *	Wilder	9A	1962	USA	LML	312	162	0.012	[2]	ERR025826‡
9L/2 *		9L	1952	USA	LML	5979	124	0.023		ERR026178‡
9L/3		9L	1941	Denmark	LML	7204	292	0.023		
9L/4	T9233/128/68	9L	1968	Denmark	LML	7240	2174	0.012	[2]	
9N/2 *		9N	1952	USA	LML	7205	None3782	0.023		ERR026179‡
9N/3		9N	1938	Denmark	LML	3983	None3782	0.023		
9N/4	533/62	9N	1962	Denmark	LML	71	66	0.023	[2]	
9N/5		9N	1962	Denmark	LML	71	66	0.012		
9N/6 *		9N	1960	Denmark	LML	71	66	0.016		ERR026180‡
9V/2		9V	1938	Denmark	LML	7200	Singleton7200	0.012		
9V/4 *	980/68	9V	1968	Denmark	LML	123	113	0.008	[2]	ERR025827‡
9V/5 *		9V	1991	Denmark	LML	162	162	0.016		ERR026181‡
9V/6 *		9V	1994	Denmark	LML	162	162	0.016		ERR026182‡
10A/1	10061/38	10A	1938	Denmark	LML	97	460	0.016	[2]	
10B/2	423/82	10B	1982	Denmark	LML	5505	Singleton5505	0.016	[2]	
10C/2	Gro Norge	10C	1983	Norway	LML	2862	Singleton2862	0.012	[2]	
10F/2 *	34355	10F	1956	USA	LML	7186	490	0.016	[2]	ERR025828‡
11A/2	1813/39	11A	1939	Denmark	LML	7214	Singleton7214	0.012	[2]	
11B/2	8087/40	11B	1940	Denmark	LML	7165	Singleton7165	0.023	[2]	
11C/1 *	Eddy nr. 53	11C	1957	USA	LML	7201	124	0.023	[2]	ERR025829‡
11D/1	70/86	11D	1986	Denmark	LML	62	62	0.023	[2]	
11F/2	34356	11F	1952	Denmark	LML	7212	None5995	0.012	[2]	
12A/2		12A	1966	Denmark	LML	5977	3122	0.012		
12A/3		12A	1966	Denmark	LML	5977	3122	0.012		

Isolate	Other Name(s)	Serotype	Year	Country	Contributor	ST	CC	Penicillin MIC	Reference(s)	Genome Accession
12A/5	559/66	12A	1966	Denmark	LML	5977	3122	0.016	[2]	
12A/6		12A	1966	Denmark	LML	5977	3122	0.016		
12A/7		12A	1966	Denmark	LML	5977	3122	0.012		
12A/8		12A	1965	Denmark	LML	5977	3122	0.012		
12A/9		12A	1966	Denmark	LML	5977	3122	0.008		
12A/10		12A	1966	Denmark	LML	5977	3122	0.012		
12A/11		12A	1966	Denmark	LML	5977	3122	0.012		
12A/12		12A	1966	Denmark	LML	5977	3122	0.012		
12A/13		12A	1966	Denmark	LML	5977	3122	0.012		
12A/14		12A	1966	Denmark	LML	5977	3122	0.012		
12B/1	Gambia 1/81	12B	1981	Gambia	LML	7187	None3745	0.064	[2]	
12F/2 *		12F	1962	USA	LML	7194	Singleton7194	0.012		ERR026183‡
12F/3 *		12F	1961	Denmark	LML	7228	4399	0.012		ERR026195‡
12F/4	6312	12F	1968	USA	LML	7194	Singleton7194	0.012	[2]	
12F/5 *		12F	1988	Denmark	LML	218	218	0.016		ERR026196‡
12F/6 *		12F	1996	Denmark	LML	218	218	0.012		ERR026187‡
13/2	34357	13	1952	USA	LML	7173	Singleton7173	0.016	[2]	
14/2 *	34359	14	1952	USA	LML	124	124	0.016	[2]	ERR025830‡
14/3 *		14	1939	Denmark	LML	875	1106	0.012		ERR026719‡
14/4 *		14	1961	Denmark	LML	134	124	0.012		ERR026188‡
14/5 *		14	1967	Denmark	LML	15	15	0.016		ERR026720‡
14/6		14	1968	Denmark	LML	7183	5401	0.008		
14/7 *		14	1992	Denmark	LML	7198	124	0.016		ERR026721‡
14/8 *		14	1976	Denmark	LML	7206	554	0.75		ERR026722‡
14/9 *		14	1982	Denmark	LML	124	124	0.008		ERR026723‡
15A/2	389/39 (da)	15A	1939	Denmark	LML	7166	Singleton7166	0.016	[2]	
15B/2	7904/39 (da)	15B	1939	Denmark	LML	1	1	0.012	[2]	
15B/3		15B	1996	Denmark	LML	2008	1	0.012		
15C/2	553/62 (da)	15C	1962	Denmark	LML	7172	1	0.023	[2]	
15F/2		15F	1952	USA	LML	7217	None5041	0.012		
15F/3	688/63 (da)	15F	1963	Denmark	LML	7177	1232	0.016	[2]	
16A/1	R105 (am)	16A	1985	USA	LML	6543	Singleton6543	0.012	[2]	
16F/2	nr.34361 (am)	16F	1952	USA	LML	7167	None659	0.016	[2]	
17A/2	nr. 4704 (ge)	17A	1971	Germany	LML	2599	2599	0.012	[2]	

Isolate	Other Name(s)	Serotype	Year	Country	Contributor	ST	CC	Penicillin MIC	Reference(s)	Genome Accession
17F/1 *	Rose	17F	1952	USA	LML	574	574	0.012	[2]	ERR025831‡
17F/2 *		17F	1939	Denmark	LML	123	113	0.012		ERR026724‡
17F/3 *		17F	1962	Denmark	LML	392	392	0.012		ERR026725‡
17F/4 *		17F	1962	Denmark	LML	392	392	0.012		ERR026726‡
18A/2	8609/43	18A	1952	Denmark	LML	241	241	0.016	[2]	
18B/2 *	1033/41 (da)	18B	1941	Denmark	LML	4706	113	0.012	[2]	ERR026193‡
18C/1	4593/40 (da)	18C	1940	Denmark	LML	4706	113	0.012	[2]	
18C/2 *		18C	1939	Denmark	LML	4706	113	0.012		ERR026186‡
18C/3 *		18C	1968	Denmark	LML	7195	113	0.008		ERR026716‡
18F/1 *	Gethens	18F	1961	USA	LML	7181	490	0.023	[2]	ERR026173‡
18F/2		18F	1945	Denmark	LML	4863	Singleton4863	0.023		
19A/2		19A	1939	Denmark	LML	7226	Singleton7226	0.016		
19A/3		19A	1952	Denmark	LML	7226	Singleton7226	0.012		
19A/5	Nr. 141/68	19A	1968	Denmark	LML	7226	Singleton7226	0.012	[2]	
19B/2	nr. 4594 (ge)	19B	1971	Germany	LML	199	199	0.032	[2]	
19C/2	7588/39 (da)	19C	1939	Denmark	LML	3785	Singleton3785	0.023	[2]	
19F/2		19F	1952	USA	LML	1	1	0.012		
19F/3	485/61 (da)	19F	1961	Denmark	LML	425	425	0.010	[2]	
19F/4		19F	1962	Denmark	LML	7229	4399	0.012		
19F/5 *		19F	1962	Denmark	LML	7229	4399	0.012		ERR026717‡
19F/6		19F	1968	USA	LML	1	1	0.012		
19F/7 *		19F	1952	Denmark	LML	7230	Singleton7230	0.016		ERR026189‡
19F/8 *		19F	1952	Denmark	LML	7229	4399	0.012		ERR026190‡
19F/10 *		19F	1963	Denmark	LML	7230	Singleton7230	0.012		ERR026174‡
19F/11 *		19F	1972	Denmark	LML	71	66	0.016		ERR026175‡
19F/12 *		19F	1995	Denmark	LML	87	87/88	0.190		ERR026185‡
19F/14		19F	1996	Spain	LML	81	81	0.380		
20/2		20	1949	USA	LML	7221	235	0.016		
20/3	34365 (am)	20	1937	Denmark	LML	235	235	0.016	[2]	
20/4		20	1962	Denmark	LML	5977	3122	0.012		
21/2		21	1952	USA	LML	7207	Singleton7207	0.016		
21/3	546/62	21	1962	Denmark	LML	193	193	0.023	[2]	
22A/2 *	3405/39(da)	22A	1939	Denmark	LML	7182	490	0.023	[2]	ERR025822‡
22A/3		22A	1939	Denmark	LML	7178	Singleton7178	0.023		

Isolate	Other Name(s)	Serotype	Year	Country	Contributor	ST	CC	Penicillin MIC	Reference(s)	Genome Accession
22F/2	1772/40 (da)	22F	1940	Denmark	LML	7219	Singleton7219	0.012	[2]	
23A/2 *	1196/45	23A	1945	Denmark	LML	439	439	0.016	[2]	ERR025821‡
23B/1	1039/41 (da)	23B	1941	Denmark	LML	7168	Singleton7168	0.016	[2]	
23B/2		23B	1996	Denmark	LML	1349	1349	0.125		
23F/2		23F	1940	Denmark	LML	7170	Singleton7170	0.016		
23F/4 *		23F	1967	Australia	LML	7184	Singleton7184	0.125	[10]	ERR026191‡
23F/5 *		23F	1979	Germany	LML	439	439	0.012		ERR026718‡
23F/6		23F	1987	Denmark	LML	81	81	1.000		
23F/10 *	Dr Melchior	23F	1996	Denmark	LML	515	439	0.023	[2]	ERR026194‡
24A/2	2748/40 (da)	24A	1940	Denmark	LML	5980	Singleton5980	0.012	[2]	
24B/2	2236/42 (da)	24B	1942	Denmark	LML	7215	Singleton7215	0.016	[2]	
24F/2	24F L (am)	24F	1952	USA	LML	7174	Singleton7174	0.016	[2]	
24F/3		24F	1943	Denmark	LML	7179	None263	0.023		
25A/1	tp 25/38; sp 65/81	25A	1981	Singapore	LML	7209	Singleton7209	0.023	[2]	
25A/2		25A	1986	Netherlands	LML	5407	5407	0.023		
25F/2		25F	1951	USA	LML	5981	Singleton5981	0.023		
25F/3		25F	1962	Denmark	LML	105	5604	0.016		
25F/4	601/62 (da)	25F	1962	Denmark	LML	105	5604	0.016	[2]	
27/2	nr. 34371 (am)	27	1956	USA	LML	1475	1475	0.032	[2]	
28A/2	1982/45 (da)	28A	1945	Denmark	LML	7225	Singleton7225	0.012	[2]	
28F/2	34372 (am)	28F	1957	USA	LML	7227	None2661	0.008	[2]	
29/2	34373 (am)	29	1952	USA	LML	5982	Singleton5982	0.023	[2]	
31/2		31	1951	USA	LML	7163	568	0.008		
31/3	nr. 34374 (am)	31	1952	USA	LML	7163	568	0.016	[2]	
31/4		31	1964	Denmark	LML	7163	568	0.012		
32A/2	2813/41(da)	32A	1941	Denmark	LML	5983	Singleton5983	0.023	[2]	
32F/2	nr. 34375 (am)	32F	1952	USA	LML	5983	Singleton5983	0.016	[2]	
33A/2	Biehl	33A	1946	USA	LML	1012	1012	0.016	[2]	
33A/3		33A	1939	Denmark	LML	1012	1012	0.012		
33B/2	E294 (da)	33B	1962	Denmark	LML	2864	Singleton2864	0.016	[2]	
33C/2	7098/41(da)	33C	1941	Denmark	LML	2865	Singleton2865	0.016	[2]	
33D/2	CSF/79	33D	1979	India	LML	2863	Singleton2863	0.023	[2]	
33D/3		33D	1979	India	LML	2863	Singleton2863	0.012		
33F/2	3084/37 (da)	33F	1937	Denmark	LML	7192	100	0.012	[2]	

Isolate	Other Name(s)	Serotype	Year	Country	Contributor	ST	CC	Penicillin MIC	Reference(s)	Genome Accession
34/2		34	1959	USA	LML	7218	Singleton7218	Unknown		
34/3		34	1968	Malaysia	LML	7202	Singleton7202	0.012		
34/4		34	1972	Denmark	LML	7235	1884	0.008		
34/5	676/74	34	1974	Denmark	LML	7216	None1041	0.008	[2]	
35A/3	1936/39 (da)	35A	1939	Denmark	LML	7242	Singleton7242	0.016	[2]	
35B/2	4356/39 (da)	35B	1939	Denmark	LML	7234	Singleton7234	0.016	[2]	
35B/3		35B	1968	Malaysia	LML	7236	Singleton7236	0.012		
35B/4		35B	1971	Denmark	LML	198	198	0.012		
35C/2 *		35C	1941	Denmark	LML	7196	113	0.012		ERR026192‡
35C/3 *	7765/43 (da)	35C	1943	Denmark	LML	5989	113	0.023	[2]	ERR025823‡
35F/2	361/39(da)	35F	1939	Denmark	LML	7164	Singleton7164	0.016	[2]	
36/2	1095/39 (da)	36	1939	Denmark	LML	7237	Singleton7237	0.012	[2]	
36/3		36	1979	USA	LML	595	None128	0.008		
36/4		36	1992	Denmark	LML	7231	Singleton7231	0.016		
36/5		36	1993	France	LML	4031	None4031	0.064		
37/2		37	1965	USA	LML	1279	447	0.016		
37/3		37	1943	Denmark	LML	447	447	0.016		
37/5		37	1972	Denmark	LML	447	447	0.012		
37/6	264/73	37	1973	Denmark	LML	7243	Singleton7243	0.012	[2]	
37/7		37	1973	USA	LML	1279	447	0.016		
37/8		37	1973	Denmark	LML	447	447	0.012		
37/9		37	1996	Denmark	LML	447	447	0.008		
38/2	9687/39 (da)	38	1939	Denmark	LML	393	393	0.012	[2]	
39/2	203/40 (da)	39	1940	Denmark	LML	7189	Singleton7189	0.012	[2]	
40/2	Coleman	40	1950	USA	LML	7185	Singleton7185	0.012	[2]	
41A/2	6803 (ge)	41A	1972	Germany	LML	7238	Singleton7238	0.016		
41A/3		41A	1972	Germany	LML	7238	Singleton7238	0.012		
41F/2	8211/40 (da)	41F	1940	Denmark	LML	7213	Singleton7213	0.012	[2]	
42/2	198/71	42	1971	Denmark	LML	433	433	0.016	[2]	
43/3	2427/48(da)	43	1948	Denmark	LML	7197	Singleton7197	0.016	[2]	
43/4		43	1954	USA	LML	7193	None5990	0.016		
44/3	Hammer	44	1956	Germany	LML	7176	Singleton7176	0.016	[2]	
45/2		45	1950	Denmark	LML	7191	Singleton7191	0.016		
45/3		45	1949	Denmark	LML	7239	Singleton7239	0.012		

Isolate	Other Name(s)	Serotype	Year	Country	Contributor	ST	CC	Penicillin MIC	Reference(s)	Genome Accession
45/4		45	1949	Denmark	LML	7191	Singleton7191	0.012		
45/5	Eddy nr.72 (am)	45	1954	USA	LML	7190	Singleton7190	0.012	[2]	
46/2	Eddy nr. 73 (am)	46	1954	USA	LML	7162	Singleton7162	0.008	[2]	
47A/1	L351	47A	1977	Unknown	LML	7220	235	0.012	[2]	
47F/1	Eddy nr. 52 (am)	47F	1977	Unknown	LML	5991	None5991	0.016	[2]	
48/2		48	1962	Denmark	LML	7233	7232	0.016		
48/3	656/63 (da)	48	1963	Denmark	LML	7232	7232	0.012	[2]	
48/4		48	1958	Denmark	LML	7232	7232	0.016		
48/5		48	1957	Denmark	LML	7232	7232	0.016		
48/6		48	1957	Denmark	LML	7232	7232	0.016		
Ala243 *	L2003-01243	14	1998	USA	ABB	13	15	1.000		ERR028708‡
Ala263 *	L2003-01263	14	2002	USA	ABB	124	124	0.023		ERR028712‡
Ala283	L2003-01283	14	2000	USA	ABB	124	124	0.008		
Ala287	L2003-01287	14	2001	USA	ABB	13	15	2.000		
Ala289 *	L2003-01289	14	2002	USA	ABB	124	124	0.016		ERR028713‡
Ala291	L2003-01291	14	1998	USA	ABB	13	15	3.000		
Ala292 *	L2003-01292	14	1998	USA	ABB	124	124	0.012		ERR028714‡
Ala317 *	L2003-01317	14	2001	USA	ABB	13	15	0.750		ERR028715‡
GE1	D105	6A	1986	Germany	RH	367	367/4247	0.250	[8]	
GE2	D106	22F	1986	Germany	RH	369	2557	0.120	[8]	
GE3	D107	23F	1986	Germany	RH	277	277	0.125	[8]	
GE4	D108	23F	1986	Germany	RH	277	277	0.500		
GE6	D110	NT	1989	Germany	RH	1106	1106	0.500		
GE7	D111	6B	1989	Germany	RH	90	90	0.750		
GE9	D113	14	1993	Germany	RH	1043	162	4.000		
GE10	D121	14	1994	Germany	RH	1043	162	0.500		
ICE7 *		7F	1997	Iceland	KGK	191	191	0.023		ERR025269‡
ICE10		12F	1998	Iceland	KGK	218	218	0.016		
ICE11 *		6B	1998	Iceland	KGK	490	490	0.012		ERR025270‡
ICE13 *		14	1998	Iceland	KGK	9	15	0.016		ERR026198‡
ICE22 *		7F	1993	Iceland	KGK	191	191	0.012		ERR028709‡
ICE23 *		7F	1993	Iceland	KGK	218	218	0.012		ERR026202‡
ICE27 *		19F	1995	Iceland	KGK	655	422/476	0.008		ERR026203‡
ICE46 *		14	2003	Iceland	KGK	124	124	0.012		ERR028710‡

Isolate	Other Name(s)	Serotype	Year	Country	Contributor	ST	CC	Penicillin MIC	Reference(s)	Genome Accession
ICE50 *		14	2003	Iceland	KGK	13	15	1.500		ERR026204[‡]
ICE57 *		9N	1994	Iceland	KGK	66	66	0.016		ERR026205[‡]
ICE186 *		18C	1996	Iceland	KGK	113	113	0.008		ERR026206[‡]
ICE211 *		23F	1999	Iceland	KGK	81	81	0.750		ERR026207[‡]
ICE269 *		18C	2005	Iceland	KGK	113	113	0.006		ERR026208[‡]
ICE501 *		18C	2002	Iceland	KGK	113	113	0.006		ERR026209[‡]
ICE570 *		14	2002	Iceland	KGK	9	15	0.016		ERR026199[‡]
ICE587		14	2002	Iceland	KGK	124	124	0.012		
ICE594 *		14	2005	Iceland	KGK	124	124	0.008		ERR026200[‡]
PMEN1 †	Spain^{23F}-1; ATCC 700669	23F	1984	Spain	LM	81	81	1.000	[3, 16]	NC_011900.1[₴]
PMEN2 *	Spain^{6B}-2; ATCC 700670	6B	1988	Spain	LM	90	90	0.500	[16]	ERR025264[‡]
PMEN3 *†	Spain^{9V}-3; ATCC 700671; SP195	9V	1993	France	LM	156	162	1.500	[16]	ABGE01000001-ABGE01000041[₴], ERR025265[‡]
PMEN4 *	Tennessee^{23F}-4; ATCC 51916	23F	1991	USA	LM	37	439	0.125	[16]	ERR025255[‡]
PMEN5 *	Spain^{14}-5; ATCC 700902	14	1990	Spain	LM	18	18	1.500	[16]	ERR025256[‡]
PMEN6	Hungary^{19A}-6; ATCC 700673	19A	1989	Hungary	LM	268	176	1.000	[16]	
PMEN7	S.Africa^{19A}-7; ATCC 700674	19A	1989	South Africa	LM	75	Singleton75	0.190	[16]	
PMEN8	S.Africa^{6B}-8; ATCC 700675	6B	1990	South Africa	LM	185	185	0.190	[16]	
PMEN9 *	England^{14}-9; ATCC 700676	14	1993	England	LM	9	15	0.016	[16]	ERR025257[‡]
PMEN10 *	CSR^{14}-10; ATCC 700677	14	1987	CSR	LM	20	15	8.000	[16]	ERR025267[‡]
PMEN11	CSR^{19A}-11; ATCC 700678	19A	1990	CSR	LM	175	Singleton175	6.000	[16]	
PMEN12 *	Finland^{6B}-12; ATCC 700903	6B	1987	Finland	LM	238	90	4.000	[16]	ERR025271[‡]
PMEN13 *	S.Africa^{19A}-13; ATCC 700904	19A	1988	South Africa	LM	41	None41	1.500	[16]	ERR028711[‡]
PMEN14 *	Taiwan^{19F}-14; ATCC 700905	19F	1997	Taiwan	LM	236	271/320	2.000	[16]	ERR026201[‡]
PMEN15 *	Taiwan^{23F}-15; ATCC 700906	23F	1997	Taiwan	LM	242	242	0.750	[16]	ERR025272[‡]
PMEN16	Poland^{23F}-16; ATCC BAA-343	23F	1995	Poland	LM	173	176	8.000	[16]	
PMEN17 *	Maryland^{6B}-17; ATCC BAA-342	6B	1997	USA	LM	384	384	1.500	[16]	ERR025273[‡]
PMEN18 *	Tennessee^{14}-18; ATCC BAA-340	14	1997	USA	LM	67	66	4.000	[16]	ERR025274[‡]
PMEN19	Columbia^{5}-19; ATCC BAA-341	5	1995	Columbia	LM	289	289	0.003	[16]	
PMEN20	Poland^{6B}-20; ATCC BAA-612	6B	1996	Poland	LM	315	315	0.120	[16]	
PMEN21	Portugal^{19F}-21; ATCC BAA-657	19F	1996	Portugal	LM	177	177	0.032	[16]	
PMEN22 *	Greece^{6B}-22; ATCC BAA-658	6B	1995	Greece	LM	273	90	0.023	[16]	ERR025275[‡]

Isolate	Other Name(s)	Serotype	Year	Country	Contributor	ST	CC	Penicillin MIC	Reference(s)	Genome Accession
PMEN23	N.Carolina^{6A}-23; ATCC BAA-659	6A	1996	USA	LM	376	2090	1.000	[16]	
PMEN24	Utah^{35B}-24; ATCC BAA-660	35B	1994	USA	LM	377	558	1.000	[16]	
PMEN25	Sweden^{15A}-25; ATCC BAA-661	15A	1998	Portugal	LM	63	63	0.120	[16]	
PMEN26	Columbia^{23F}-26; ATCC BAA-662	23F	1998	Portugal	LM	338	176	0.120	[16]	
PMEN27	Sweden^{1}-27; ATCC BAA-1654	1	1992	Sweden	LM	217	217	0.006		
PMEN28	Sweden^{1-}28	1	1997	Sweden	LM	306	306	0.006		
PMEN29	USA^{1}-29	1	1948	USA	LM	615	615	0.012		
PMEN30	Greece^{21}-30	21	1996	Greece	LM	193	193	0.016		
PMEN31	Netherlands^{3}-31	3	1984	Netherlands	LM	180	180	0.023		
PMEN32	Denmark^{14}-32	14	1996	Denmark	LM	230	230	1.000		
PMEN33	Netherlands^{8}-33	8	1989	Netherlands	LM	53	62	0.016		
PMEN34 *	Denmark^{12F}-34	12F	1995	Canada	LM	218	218	0.016		ERR025276‡
PMEN35 *	Netherlands^{14}-35	14	1980	Netherlands	LM	124	124	0.016		ERR025277‡
PMEN36 *	Netherlands^{18C}-36	18C	1980	Netherlands	LM	113	113	0.023		ERR025278‡
PMEN37	Netherlands^{15B}-37	15B	1987	Netherlands	LM	199	199	0.012		
PMEN38	Sweden^{4}-38	4	1995	Sweden	LM	205	205	0.012		
PMEN39 *	Netherlands^{7F}-39	7F	1984	Netherlands	LM	191	191	0.012		ERR025268‡
PMEN40	Sweden^{1}-40	1	1995	Sweden	LM	304	304	0.012		
PMEN41	Portugal^{6A}-41	6A	1996	Portugal	LM	327	395	0.012		
PMEN42	Norway^{NT}-42	NT	1996	Portugal	LM	344	344	0.094		
PMEN43	USA^{NT}-43	NT	1980	USA	LM	448	448	0.094		
PN1	Pn12	6	1972	PNG	RH	7151	277	1.000	[13]	
PN2	P1	4	1969	PNG	RH	7152	Singleton7152	1.000	[6]	
SA1	203A	19A	1978	South Africa	MRJ	41	None41	4.000	[12, 19]	
SA2	223 (19A)	19A	1978	South Africa	MRJ	41	None41	2.000	[12, 19]	
SA3	381	19A	1978	South Africa	MRJ	41	None41	4.000	[12, 19]	
SA4	107	19A	1978	South Africa	MRJ	41	None41	4.000	[12, 19]	
SA5	61857	19A	1978	South Africa	MRJ	41	None41	2.000	[12, 19]	
SA6	61783	19A	1978	South Africa	MRJ	41	None41	2.000	[12, 19]	
SA7	145	19A	1978	South Africa	MRJ	1094	1094	>4.000	[12, 19]	
SA8	209	19A	1978	South Africa	MRJ	1605	None41	4.000	[12, 19]	
SA9	205	19A	1978	South Africa	MRJ	1605	None41	4.000	[12, 19]	

Isolate	Other Name(s)	Serotype	Year	Country	Contributor	ST	CC	Penicillin MIC	Reference(s)	Genome Accession
SA10	DURBANT	19A	1977	South Africa	MRJ	1656	None68	4.000	[1, 19]	
SA11	60292	6A	1978	South Africa	MRJ	1094	1094	4.000	[12, 19]	
SA12	994	6A	1978	South Africa	MRJ	1094	1094	4.000	[12, 19]	
SA13	60293	6A	1978	South Africa	MRJ	1094	1094	2.000	[12, 19]	
SA14	62272	6A	1978	South Africa	MRJ	1094	1094	4.000	[12, 19]	
SA15	532	6A	1978	South Africa	MRJ	1094	1094	2.000	[12, 19]	
SA16	61155	6A	1978	South Africa	MRJ	1094	1094	4.000	[12, 19]	
SA17	13811	6A	1978	South Africa	MRJ	1094	1094	4.000	[12, 19]	
SA18	223 (6A)	6A	1978	South Africa	MRJ	1094	1094	2.000	[12, 19]	
SA19	61153	6A	1978	South Africa	MRJ	1094	1094	4.000	[12, 19]	
SA20	61154	6A	1978	South Africa	MRJ	1094	1094	4.000	[12, 19]	
SA21	135	6A	1978	South Africa	MRJ	1607	1094	4.000	[12, 19]	
SA22	61855	14	1978	South Africa	MRJ	124	124	0.250	[12, 19]	
SA23	61370	19A	1978	South Africa	MRJ	172	176	0.500	[12, 19]	
SA24	A922	19A	1978	South Africa	MRJ	1608	None1608	0.250	[12, 19]	
SA25	A910	19A	1978	South Africa	MRJ	1608	None1608	0.500	[12, 19]	
SA26	A1002	19A	1978	South Africa	MRJ	1610	None1608	0.500	[12, 19]	
SA27	SA4; 8256	14	1978-1980	South Africa	RH	5174	15	0.023	[8]	
SA28	SA5; 272	14	1978-1980	South Africa	RH	5174	15	0.012	[8]	
SA31	SA8; 2039	6A	1978-1980	South Africa	RH	1094	1094	2.000	[8]	
SA32	SA9; 10760	6A	1978-1980	South Africa	RH	1094	1094	4.000	[8]	
SA34	SA11; 11405	29	1988	South Africa	RH	5178	None5177	0.015		
SA35	SA12; 45607	29	1987	South Africa	RH	5178	None5177	0.030	[8]	
SA36	SA13; 60814	29	1987	South Africa	RH	5178	None5177	0.250	[8, 9]	
SA37	SA14; 56828	19F	1987	South Africa	RH	5179	Singleton5179	0.120	[8]	
SA38	SA18; 60785	6A	1987	South Africa	RH	1094	1094	16.000	[8]	
SA39	SA19; 63509	19A	1987	South Africa	RH	41	None41	0.750	[8]	
SA40	SA20; 205	19A	mid 1980s	South Africa	RH	41	None41	1.000		
SA41	SA21; 215	6B	mid 1980s	South Africa	RH	1094	1094	0.500		
SA42	SA22; 613	19F	mid 1980s	South Africa	RH	763	271/320	0.008		
SA43	SA23; 20463	19A	mid 1980s	South Africa	RH	41	None41	0.250		
SA44	SA24; 39062	6A	mid 1980s	South Africa	RH	1094	1094	2.000		
SA45	SA25; 60292	6A	mid 1980s	South Africa	RH	1094	1094	4.000		
SA47	97-1	19A	1979	South Africa	KPK, AVG	1605	None41	2.000		

Isolate	Other Name(s)	Serotype	Year	Country	Contributor	ST	CC	Penicillin MIC	Reference(s)	Genome Accession
SA48	48	19A	1979	South Africa	KPK, AVG	1656	None68	4.000		
SA49	48-2	19A	1982	South Africa	KPK, AVG	7255	Singleton7255	0.023		
SA51	46878	6B	1989	South Africa	KPK, AVG	185	185	0.125		
SA52	13979	6B	1984	South Africa	KPK, AVG	2421	2421/2909	0.250		
SA53	N383	11A	1983	South Africa	KPK, AVG	7247	1010	0.250		
SA54	136664	6B	1983	South Africa	KPK, AVG	7084	None5431	0.125		
SA55	31664	19A	1983	South Africa	KPK, AVG	75	Singleton75	0.250		
SA56	6415	6B	1984	South Africa	KPK, AVG	1094	1094	2.000		
SA57	108551	6B	1984	South Africa	KPK, AVG	185	185	0.125		
SA58	19931	19F	1986	South Africa	KPK, AVG	7249	Singleton7249	0.125		
SA59	3630	6A	1985	South Africa	KPK, AVG	7251	1094	0.250		
SA60	511	19A	1985	South Africa	KPK, AVG	172	176	0.125		
SA61	97	19A	1985	South Africa	KPK, AVG	5183	176	0.125		
SA62	28649	6B	1985	South Africa	KPK, AVG	1094	1094	1.000		
SA63	93343	14	1985	South Africa	KPK, AVG	124	124	0.125		
SA64	6482	19F	1985	South Africa	KPK, AVG	7254	Singleton7254	0.125		
SA65	6076	38	1985	South Africa	KPK, AVG	7252	Singleton7252	0.250		
SA66	6428	6B	1985	South Africa	KPK, AVG	185	185	0.125		
SA67	34023	6B	1985	South Africa	KPK, AVG	7250	Singleton7250	0.125		
SA68	03904	6A	1985	South Africa	KPK, AVG	7257	1094	0.500		
SA69	34142	19A	1985	South Africa	KPK, AVG	7248	Singleton7248	0.500		
SA70	6130	19A	1985	South Africa	KPK, AVG	5183	176	0.250		
SA71	34581	23F	1989	South Africa	KPK, AVG	6215	81	0.250		
SA72	34139	6A	1988	South Africa	KPK, AVG	1094	1094	1.000		
SA73	48628	19A	1988	South Africa	KPK, AVG	41	None41	4.000		
SA74	13897	23F	1988	South Africa	KPK, AVG	6215	81	1.000		
SA75	13619	23F	1987	South Africa	KPK, AVG	6215	81	0.500		
SA76	95103	14	1988	South Africa	KPK, AVG	7246	Singleton7246	0.125		
SA77	26940	23F	1987	South Africa	KPK, AVG	6215	81	1.000		
SA78	47590	14	1989	South Africa	KPK, AVG	7253	Singleton7253	0.250		
SA79	49171	19A	1989	South Africa	KPK, AVG	172	176	0.125		
SA80	22453	23F	1989	South Africa	KPK, AVG	81	81	0.500		
SA81	23099	23F	1989	South Africa	KPK, AVG	6215	81	0.500		
SA82	47085	14	1989	South Africa	KPK, AVG	U	124	0.250		

Isolate	Other Name(s)	Serotype	Year	Country	Contributor	ST	CC	Penicillin MIC	Reference(s)	Genome Accession
SA83	46518	6B	1989	South Africa	KPK, AVG	53	62	0.047		
SA84	SA3; 35897	14	1978-1980	South Africa	RH	5174	15	0.060	[9]	
SN1	602	6B	1987	Spain	JL	90	90	0.250	[7, 17, 18]	
SN2	629	9V	1987	Spain	JL	156	162	1.500		
SN3	795	9V	1988	Spain	JL	156	162	0.750	[21]	
SN4	946	9V	1988	Spain	JL	156	162	2.000	[22]	
SN5	998	9V	1988	Spain	JL	156	162	2.000		
SN6	1196	6B	1989	Spain	JL	3999	90	1.500		
SN7	1064	6B	1989	Spain	JL	7161	90	1.000		
SN8	1070	9V	1989	Spain	JL	156	162	2.000		
SN9	1000	9V	1989	Spain	JL	156	162	1.000		
USA1	4793-00	18C	1999	USA	LM, BWB	113	113	<=0.03		
USA2 *	6223-99	18C	1999	USA	LM, BWB	113	113	<=0.03		ERR028716‡
USA3	6543-99	18C	1999	USA	LM, BWB	113	113	<=0.03		
USA4	0050-02	14	2001	USA	LM, BWB	124	124	<=0.03		
USA5	6605-01	14	2001	USA	LM, BWB	124	124	<=0.03		
USA6 *	4347-99	14	1999	USA	LM, BWB	124	124	<=0.03		ERR025254‡
USA7	2836-99	14	1999	USA	LM, BWB	124	124	<=0.03		
USA8 *	0680-02	9N	2001	USA	LM, BWB	8119	66	<=0.03		ERR025258‡
USA9 *	PATH#183	14	1999	Brazil	LM, BWB	66	66	0.094		ERR025259‡
USA10	PATH#184	14	1999	Brazil	LM, BWB	66	66	0.125		
USA11 *	PATH#286	14	Unknown	Brazil	LM, BWB	66	66	0.064		ERR025260‡
USA12 *	PATH#678	23F	2001	USA	LM, BWB	66	66	<=0.03		ERR025261‡
USA13 *	PATH#688	19F	2005	Germany	LM, BWB	66	66	<=0.03		ERR025262‡
USA14	1087-00	7F	1999	USA	LM, BWB	191	191	<=0.03		
USA15	7102-99	7F	1999	USA	LM, BWB	191	191	<=0.03		
USA16 *	PATH#224	7F	2003	Brazil	LM, BWB	191	191	0.004		ERR028717‡
USA17	2008008776	7F	2005	USA	LM, BWB	191	191	0.016		
USA18 *	4621-00	12F	1999	USA	LM, BWB	218	218	<=0.03		ERR028718‡
USA19	5400-00	7F	1999	USA	LM, BWB	218	218	<=0.06		
USA20 *	PATH#129	7F	1999	South Africa	LM, BWB	218	218	<=0.03		ERR025263‡
USA21	PATH#143	6A	2007	Turkey	LM, BWB	490	490	<=0.03		
USA22 *	3140-06	6C	2005	USA	LM, BWB	490	490	0.500		ERR028719‡
USA23	89FL23F	23F	1989	USA	LM	33	439	2.000	[15]	

Isolate	Other Name(s)	Serotype	Year	Country	Contributor	ST	CC	Penicillin MIC	Reference(s)	Genome Accession
USA24	89SA23F	23F	1989	South Africa	LM	81	81	4.000	[15]	
USA25	90MI14	14	1990	USA	LM	7245	None22	2.000	[15]	
USA26	85PA23F	23F	1985	USA	LM	81	81	4.000	[15]	
USA27	85NY23F	23F	1985	USA	LM	1906	439	<=0.03	[15]	
USA28	88FL23F	23F	1988	USA	LM	81	81	2.000	[15]	
USA29	90GA14	14	1990	USA	LM	18	18	2.000	[15]	
USA30	91NC23F	23F	1991	USA	LM	81	81	2.000	[15]	
USA31	83MI23F	23F	1983	USA	LM	7256	Singleton7256	1.000	[15]	
USA32	91AZ23F	23F	1991	USA	LM	81	81	2.000	[15]	
USA33	81CO6B	6B	1981	USA	LM	238	90	1.000	[15]	
USA34	85NY19A	19A	1985	USA	LM	41	None41	8.000	[15]	
USA35	89NY23F	23F	1989	USA	LM	81	81	4.000	[15]	
USA36	90OH23F	23F	1990	USA	LM	81	81	2.000	[15]	
USA37	91MT23F	23F	1991	USA	LM	81	81	4.000	[15]	
USA38	90TX23F	23F	1990	USA	LM	81	81	4.000	[15]	
USA39	90MO6B	6B	1990	USA	LM	90	90	2.000	[15]	
USA40	90OK23F	23F	1990	USA	LM	81	81	2.000	[15]	
USA41	90IN23F	23F	1990	USA	LM	81	81	4.000	[15]	
USA42	84PA23F	23F	1984	USA	LM	81	81	4.000	[15]	
USA43	90NB23F	23F	1990	USA	LM	81	81	4.000	[15]	
USA44	88AZ23F	23F	1988	USA	LM	81	81	2.000	[15]	
USA45	91CA23F	23F	1991	USA	LM	81	81	2.000	[15]	
USA46	89NB23F	23F	1989	USA	LM	81	81	4.000	[15]	
USA47	85UT23F	23F	1985	USA	LM	81	81	2.000	[15]	
USA48	91GA23F	23F	1991	USA	LM	37	439	1.000	[15]	
USA49	89CA23F	23F	1989	USA	LM	81	81	4.000	[15]	
670 †		6B	1988	Spain		90	90	Unknown		CP002176.1[₴]
70585 †		5	Unknown	Unknown		289	289	Unknown		CP000918.1[₴]
CDC0288-04 †		12F	2003-2004	USA		220	218	Unknown		ABGF01000001-ABGF01000038[₴]
CDC1087-00 †		7F	1999	USA		191	191	0.030		ABFT01000001-ABFT01000075[₴]
CDC1873-00 †		6A	1999	USA		376	2090	4.000		ABFS01000001-ABFS01000053[₴]
CDC3059-06 †		19A	2005	USA		199	199	0.120		ABGG01000001-ABGG01000033[₴]
CGSP14 †		14	2004-2005	Taiwan		15	15	2.000	[4]	NC_010582.1[₴]
D39 †		2	1916	Unknown		595	None128	Unknown	[14]	CP000410.1[₴]

Isolate	Other Name(s)	Serotype	Year	Country	Contributor	ST	CC	Penicillin MIC	Reference(s)	Genome Accession
JJA †		14	1995	Brazil		66	66	I	[5]	CP000919.1ƻ
MLV-016 †		11A	Unknown	Unknown		62	62	Unknown		ABGH01000001-ABGH01000126ƻ
P1031 †		1	2002	China		303	217	S		CP000920.1ƻ
R6 †		NT	1964	Unknown		595	None128	Unknown	[11]	AE007317.1ƻ
SPnINV104 †		1	1998	England		227	306	S	[5]	FQ312030.1ƻ
SPnINV200 †		14	1995	England		9	15	S	[5]	FQ312029.1ƻ
SPnOXC141 †		3	2001	England		180	180	0.012	[5]	FQ312027.1ƻ
TIGR4 †		4	1991	Norway		205	205	Unknown	[20]	AE005672.3ƻ

* Isolate selected for whole-genome sequencing in this study. † Genome retrieved from Genbank. ‡ European Nucleotide Archive accession. ƻ Genbank accession. Abbreviations: USA = United States of America; CSR = Czech and Slovak Federal Republic; PNG = Papua New Guinea; LML = Lotte Munch Lambertsen; ABB = Angela B Brueggemann; RH = Regine Hakenbeck; KGK = Karl G Kristinsson; LM = Lesley McGee; MRJ = Michael R Jacobs; KPK = Keith P Klugman; AVG = Anne von Gottberg; JL = Josefina Liñares; BWB = Bernard W Beall; ST = sequence type; CC = clonal complex; MIC = Minimum inhibitory concentration (µg/ml); S = susceptible; I = intermediately resistant.

References

1. **Appelbaum, P. C., J. N. Scragg, A. J. Bowen, A. Bhamjee, A. F. Hallett, and R. C. Cooper.** 1977. *Streptococcus pneumoniae* resistant to penicillin and chloramphenicol. Lancet **2:**995-997.
2. **Bentley, S. D., D. M. Aanensen, A. Mavroidi, D. Saunders, E. Rabbinowitsch, M. Collins, K. Donohoe, D. Harris, L. Murphy, M. A. Quail, G. Samuel, I. C. Skovsted, M. S. Kaltoft, B. Barrell, P. R. Reeves, J. Parkhill, and B. G. Spratt.** 2006. Genetic analysis of the capsular biosynthetic locus from all 90 pneumococcal serotypes. PLoS Genet. **2:**262-269.
3. **Croucher, N. J., D. Walker, P. Romero, N. Lennard, G. K. Paterson, N. C. Bason, A. M. Mitchell, M. A. Quail, P. W. Andrew, J. Parkhill, S. D. Bentley, and T. J. Mitchell.** 2009. Role of conjugative elements in the evolution of the multidrug-resistant pandemic clone *Streptococcus pneumoniae*^{Spain23F} ST81. J Bacteriol. **191:**1480-1489.
4. **Ding, F., P. Tang, M.-H. Hsu, P. Cui, S. Hu, J. Yu, and C.-H. Chiu.** 2009. Genome evolution driven by host adaptations results in a more virulent and antimicrobial-resistant *Streptococcus pneumoniae* serotype 14. BMC Genet. **10:**158.
5. **Donati, C., N. L. Hiller, H. Tettelin, A. Muzzi, N. J. Croucher, S. V. Angiuoli, M. Oggioni, J. C. Dunning Hotopp, F. Z. Hu, D. R. Riley, A. Covacci, T. J. Mitchell, S. D. Bentley, M. Kilian, G. D. Ehrlich, R. Rappuoli, E. R. Moxon, and V. Masignani.** 2010. Structure and dynamics of the pan-genome of *Streptococcus pneumoniae* and closely related species. Genome Biol. **11:**R107.
6. **Dowson, C. G., A. Hutchison, J. A. Brannigan, R. C. George, H. David, J. Liñares, A. Tomasz, J. M. Smith, and B. G. Spratt.** 1989. Horizontal transfer of penicillin-binding protein genes in penicillin-resistant clinical isolates of *Streptococcus pneumoniae*. Proc Natl Acad Sci U S A. **86:**8842-8846.
7. **Fernández, A., C. Cabellos, F. Tubau, J. Liñares, P. F. Viladrich, and F. Gudiol.** 2001. Relationship between penicillin and cephalosporin resistance of *Streptococcus pneumoniae* strains and its inflammatory activity in the experimental model of meningitis. Med Microbiol Immunol. **190:**135-138.
8. **Hakenbeck, R., T. Briese, L. Chalkley, H. Ellerbrok, R. Kalliokoski, C. Latorre, M. Leinonen, and C. Martin.** 1991. Antigenic variation of penicillin-binding proteins from penicillin-resistant clinical strains of *Streptococcus pneumoniae.* J Infect Dis. **164:**313-319.
9. **Hakenbeck, R., T. Briese, L. Chalkley, H. Ellerbrok, R. Kalliokoski, C. Latorre, M. Leinonen, and C. Martin.** 1991. Variability of penicillin-binding proteins from penicillin-sensitive *Streptococcus pneumoniae*. J Infect Dis. **164:**307-312.
10. **Hansman, D., and M. M. Bullen.** 1967. A resistant pneumococcus. Lancet **2:**264-265.
11. **Hoskins, J., W. E. Alborn, J. Arnold, L. C. Blaszczak, S. Burgett, B. S. DeHoff, S. T. Estrem, L. Fritz, D. J. Fu, W. Fuller, C. Geringer, R. Gilmour, J. S. Glass, H. Khoja, A. R. Kraft, R. E. Lagace, D. J. LeBlanc, L. N. Lee, E. J. Lefkowitz, J. Lu, P. Matsushima, S. M. McAhren, M. McHenney, K. McLeaster, C. W. Mundy, T. I. Nicas, F. H. Norris, M. O'Gara, R. B. Peery, G. T. Robertson, P. Rockey, P. M. Sun, M. E. Winkler, Y. Yang, M. Young-Bellido, G. S. Zhao, C. A. Zook, R. H. Baltz, S. R. Jaskunas, P. R. Rosteck, P. L. Skatrud, and J. I. Glass.** 2001. Genome of the bacterium *Streptococcus pneumoniae* strain R6. J Bacteriol. **183:**5709-5717.
12. **Jacobs, M. R., H. J. Koornhof, R. M. Robins-Browne, C. M. Stevenson, Z. A. Vermaak, I. Freiman, B. Miller, M. A. Witcomb, M. Isaäcson, J. I. Ward, and R. Austrian.** 1978. Emergence of multiply resistant pneumococci. N Engl J Med. **299:**735-740.
13. **Laible, G., B. G. Spratt, and R. Hakenbeck.** 1991. Interspecies recombinational events during the evolution of altered PBP 2x genes in penicillin-resistant clinical isolates of *Streptococcus pneumoniae*. Mol Microbiol. **5:**1993-2002.

14. **Lanie, J. A., W. L. Ng, K. M. Kazmierczak, T. M. Anrzejewski, T. M. Davidson, K. J. Wayne, H. Tettelin, J. I. Glass, and M. E. Winkler.** 2007. Genome sequence of Avery's virulent serotype 2 strain D39 of *Steptococcus pneumoniae* and comparison with that of unencapsulated laboratory strain R6. J Bacteriol. **189:**38-51.
15. **McDougal, L. K., J. K. Rasheed, J. W. Biddle, and F. C. Tenover.** 1995. Identification of multiple clones of extended-spectrum cephalosporin-resistant *Streptococcus pneumoniae* isolates in the United States. Antimicrob Agents Chemother. **39:**2282-2288.
16. **McGee, L., L. McDougal, J. Zhou, B. G. Spratt, F. C. Tenover, R. George, R. Hakenbeck, W. Hryniewicz, J. C. Lefévre, A. Tomasz, and K. P. Klugman.** 2001. Nomenclature of major antimicrobial-resistant clones of *Streptococcus pneumoniae* defined by the Pneumococcal Molecular Epidemiology Network. J Clin Microbiol. **39:**2565-2571.
17. **Muñoz, R., C. G. Dowson, M. Daniels, T. J. Coffey, C. Martin, R. Hakenbeck, and B. G. Spratt.** 1992. Genetics of resistance to third-generation cephalosporins in clinical isolates of *Streptococcus pneumoniae*. Mol Microbiol. **6:**2461-2465.
18. **Pallarés, R., J. Liñares, M. Vadillo, C. Cabellos, F. Manresa, P. F. Viladrich, R. Martin, and F. Gudiol.** 1995. Resistance to penicillin and cephalosporin and mortality from severe pneumococcal pneumonia in Barcelona, Spain. N Engl J Med. **333:**474-480.
19. **Reinert, R. R., J. M. R, P. C. Appelbaum, S. Bajaksouzian, S. Cordeiro, M. van der Linden, and A. Al-Lahham.** 2005. Relationship between the original multiply resistant South African isolates of *Streptococcus pneumoniae* from 1977 to 1978 and contemporary international resistant clones. J Clin Microbiol. **43:**6035-6041.
20. **Tettelin, H., K. E. Nelson, I. T. Paulsen, J. A. Eisen, T. D. Read, S. Peterson, J. Heidelberg, R. T. DeBoy, D. H. Haft, R. J. Dodson, A. S. Durkin, M. Gwinn, J. F. Kolonay, W. C. Nelson, J. D. Peterson, L. A. Umayam, O. White, S. L. Salzberg, M. R. Lewis, D. Radune, E. Holtzapple, H. Khouri, A. M. Wolf, T. R. Utterback, C. L. Hansen, L. A. McDonald, T. V. Feldblyum, S. Angiuoli, T. Dickinson, E. K. Hickey, I. E. Holt, B. J. Loftus, F. Yang, H. O. Smith, J. C. Venter, B. A. Dougherty, D. A. Morrison, S. K. Hollingshead, and C. M. Fraser.** 2001. Complete genome sequence of a virulent isolate of *Streptococcus pneumoniae.* Science **293:**498-506.
21. **Viladrich, P. F., C. Cabellos, R. Pallarés, F. Tubau, J. Martinez-Lacasa, J. Liñares, and F. Gudiol.** 1996. High doses of cefotaxime in treatment of adult meningitis due to *Streptococcus pneumoniae* with decreased susceptibilities to broad spectrum cephalosporins. Antimicrob Agents Chemother. **40:**218-220.
22. **Viladrich, P. F., F. Gudiol, J. Liñares, R. Pallarés, I. Sabaté, G. Rufí, and J. Ariza.** 1991. Evaluation of vancomycin for therapy of adult pneumococal meningitis. Antimicrob Agents Chemother. **35:**2467-2472.
