# Supplementary material for: The multidrug-resistant PMEN1 pneumococcus is a paradigm for genetic success
Source: Genome Biol. 2012 Nov 16;13(11):R103. doi: 10.1186/gb-2012-13-11-r103 (PMC3580495; doi:10.1186/gb-2012-13-11-r103)
Supplement: Additional file 9 — Figure S3. A variable site map of one PMEN3 putative imported region and flanking regions. [file gb-2012-13-11-r103-S9.PDF]

```
                                1111122222 2222222222 2222233333 3333333333 3333344444
      3444444556 6666666679 0333400468 8888888999 9999900000 0011112233 3333414555
      7345667351 2256788809 6669424121 1378899012 5667744456 6755573711 1167394189
      3265476314 0398403958 8035356281 7511567432 9271637842 6303671735 6779204087
PMEN3 CCTAAACCAC TTTGCGACAA AGACGCAATC TCGGAACTGT TGTAAAACGT CTGCCTCTGG TTGTGTTAAC
9A/1  .......... .......... .CGTATGGCT CTAACGACTC CACTGGCAAC TCATTCTCAA AATCACCGGT
9V/5  .......... .......... .CGTA-GGCT CTAAC.ACTC CACTGGCAAC TCATTCTCAA AATCACCGGT
9V/6  .......... .......... .CGTATGGCT CTAAC.ACTC CACTGGCAAC TCATTCTCAA AATCACCGGT
PMEN1 TTCTGGAAGT GACATATTGG G......... .......... .......... .......... ..........

      4555555555 5555555555 5555666666 6666666666 6777777777 7777777777 7788888899
      8233444457 7788889999 9999113344 4445559999 9000000011 1111111222 2215888923
      7556235985 7902771234 5569172334 4480032388 9011223324 4467778134 4655699347
      3640074361 8383486903 3760086510 3681337647 9824692350 3640392932 5870639581
PMEN3 TTGTACGTTT GGTTAAAGCA CGTCCTATAC ATTGATTAAA CTTAATCATC ACGTTAGGCA AAGCGATGGC
9A/1  CCACGTAGCC AACGCCGAGC ATCATCCCTT TCGATCCCTT TCCCTCACGT GAACCTAATT GGT.......
9V/5  CCACGTAGCC AACGCCGAGC ATCATCCCTT TCGATCCCTT TCCCTCACGT GAACCTAATT GGT.......
9V/6  CCACGTAGCC AACGCCGAGC ATCATCCCTT TCGATCCCTT TCCCTCACGT GAACCTAATT GGT.......
PMEN1 .......... .......... .......... .......... .......... .......... ...TAGCTAA
```

Figure S3. Variable site output showing the PMEN3 putative import region 4 and flanking regions. Regions from the genomes of the PMEN1 reference strain, the PMEN3 reference strain and the PMEN3 ancestral representatives, 9A/1, 9V/5 and 9V/6 are shown. Numbers indicate variable site positions within the alignment. Periods (".") indicate bases identical to the PMEN3 reference at a given site. Dashes ("-") indicate missing bases at a given site. Red brackets indicate the putative imported region.
